# Targeted Intervention Strategies for Maternal–Offspring Transmission of *Christensenellaceae* in Pigs via a Deep Learning Model

**DOI:** 10.1002/advs.202503411

**Published:** 2025-06-10

**Authors:** Haibo Shen, Xiaokang Ma, Longlin Zhang, Hao Li, Jichang Zheng, Shengru Wu, Ke Zuo, Yulong Yin, Jing Wang, Bie Tan

**Affiliations:** ^1^ Key Laboratory of Hunan Province for the Products Quality Regulation of Livestock and Poultry College of animal science and technology Hunan Agricultural University Changsha 410128 China; ^2^ Yuelushan Laboratory Hunan 410128 China; ^3^ College of Animal Science and Technology Northwest A&F University Yangling Shaanxi 712100 China; ^4^ National & Local Joint Engineering Research Center of Targeted and Innovative Therapeutics Chongqing Key Laboratory of Kinase Modulators as Innovative Medicine College of Pharmacy (International Academy of Targeted Therapeutics and Innovation) Chongqing University of Arts and Sciences Chongqing 402160 China; ^5^ Laboratory of Animal Nutritional Physiology and Metabolic Process Key Laboratory of Agro‐ecological Processes in Subtropical Region National Engineering Laboratory for Pollution Control and Waste Utilization in Livestock and Poultry Production Institute of Subtropical Agriculture Chinese Academy of Sciences Changsha 410125 China

**Keywords:** *Christensenella minuta*, deep learning, early life microbial development, gut microbiota, maternal microbial transmission, Ningxiang pigs, pig microbiome

## Abstract

Understanding the mechanisms of maternal microbial transmission is crucial for early gut microbiota development and long‐term health outcomes in offspring. However, early maternal microbial interventions remain a challenge due to the complexity of accurately identifying transmitted taxa. Here, the maternal–offspring microbial transmission model (MOMTM), a deep learning framework specifically designed to map maternal microbiota transmission dynamics across pig breeds and developmental stages, is introduced. Using MOMTM, key transmitted taxa, such as the *Christensenellaceae R‐7* are successfully predicted, which show high transmission centrality during early development periods. Additionally, it is demonstrated that galacto‐oligosaccharide intervention in sows promotes a *Christensenellaceae R‐7*‐dominated enterotype and improves fiber digestibility in offspring. Further analysis reveals that *Christensenellaceae*, particularly *Christensenella minuta*, have enhanced adhesion and mucin utilization capabilities, facilitating its gut colonization. These findings highlight MOMTM's potential as a novel approach for microbiota‐targeted health interventions in early life, offering insights into strategies that promote gut health and development from birth.

## Graphical abstract

This study deciphers maternal‐driven microbial transmission in pigs using a deep learning model (maternal–offspring microbial transmission model), identifying *Christensenellaceae R‐7* group as a key transmitted taxon. Filtered galacto‐oligosaccharides‐targeted *Christensenellaceae R‐7* group alters offspring microbial functions via maternal supplementation. Functional assays reveal enhanced adhesion and mucin utilization by *Christensenella minuta*, in mice. The work enables targeted microbiome interventions for improving offspring health.

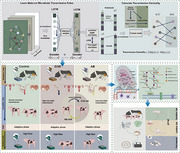

## 1 Introduction

The early establishment of the gut microbiota is crucial for host development, impacting immune maturation, nutrient metabolism, and risk of diseases.^[^ ^1^, ^2^, ^3^, ^4^, ^5^, ^6^ ^]^ Initial colonization, which is shaped by factors such as delivery mode and feeding practices, gradually stabilizes into an adult‐like community.^[^ ^7^ ^]^ Vertical transmission, the transfer of microbiota from mother to offspring, is central to the initial colonization of the infant,^[^ ^8^ ^]^ although the question of whether microbial colonization of the gut begins in utero remains a topic of ongoing debate.^[^ ^9^ ^]^ Maternal diet influences both the mother's and infant's microbiomes, with probiotics reducing infant allergies and sweeteners increasing offspring body fat and disrupting gut microbiota.^[^ ^10^, ^11^ ^]^ Gavage of fetal mice with human vaginal microbiota affects offspring metabolism, immunity, and brain development.^[^ ^12^ ^]^ Despite these promising findings, assessing the dynamics of maternal microbial transmission and developing strategies to precisely regulate this transfer remain significant challenges. Longitudinal sampling is commonly used to study vertical transmission of microbiome species, strains, and genes, often through high‐resolution metagenomic sequencing and Single Nucleotide Variation (SNV) analysis.^[^ ^8^ ^]^ While studies like Yassour et al.^[^ ^13^ ^]^ and Valles‐Colomer et al.^[^ ^14^ ^]^ have tracked microbial transmission, these approaches struggle to clarify whether shared strains are transmitted directly from mother to offspring or acquired from the environment. Moreover, the accuracy of SNV analysis is highly dependent on the availability of representative reference genomes. Traditional human‐derived reference databases, which are extensively applied in these analyses, may not fully capture the microbial diversity and strain‐specific variations present in nonhuman hosts (e.g., livestock such as pigs). This reference bias can lead to missed or inaccurate SNV detection, further complicating the dissection of transmission dynamics. Similarly, compositional source‑tracking tools like FEAST^[^ ^15^ ^]^ and SourceTracker^[^ ^16^ ^]^ yield only static estimates of maternal contribution and ignore both temporal succession and host–microbe crosstalk. This limitation complicates the accurate evaluation of the broader impact of these variables on microbial transmission and targeted maternal microbiota interventions.

Machine learning (ML) models, especially long short‐term memory (LSTM) models, are powerful tools for analyzing complex microbial transmission dynamics.^[^ ^17^, ^18^, ^19^, ^20^, ^21^, ^22^ ^]^ The gut microbiome evolves dynamically from birth to adulthood, influenced by numerous factors.^[^ ^23^ ^]^ ML can uncover relationships between microbial communities and environmental or host factors, while LSTMs excel at capturing temporal dependencies in longitudinal data, predicting future microbial trajectories, and differentiating transmission sources. Attention mechanisms, like those used in LSTM and BERT models, further enhance the ability to identify key patterns and prioritize important features in large‐scale microbiome data.^[^ ^24^ ^]^ Pigs are ideal models for studying these shifts due to their physiological and immunological similarities to humans, well‐defined genetics, and controlled environments.^[^ ^25^, ^26^ ^]^ The attention‐based LSTM model holds significant potential for analyzing longitudinal microbiome data under the influence of multiple factors, offering distinct advantages in capturing complex, dynamic interactions. This capability could have profound implications for microbial interventions and therapeutic applications, enabling more precise and context‐aware strategies for microbiome modulation.

In this study, we present the maternal–offspring microbial transmission model (MOMTM), a first attention‐based deep learning framework designed to predict and analyze maternal microbial transmission dynamics in pigs. MOMTM integrates microbiome data from different pig breeds, environment, and diet to capture the maternal microbial transmission. Interestingly, the *Christensenellaceae R‐7* group was identified as a key maternal taxon with high transmission centrality during critical lactation windows by using this model. Based on these predictions, we show that targeted maternal microbial intervention with galacto‐oligosaccharides (GOS) enhanced the establishment of a *Christensenellaceae R‐7*‐group‐dominated enterotype and improved fiber digestibility in offspring. Notably, *Christensenellaceae*, particularly *Christensenella minuta*, possess a competitive advantage in adhesion and mucin utilization, aiding their gut colonization. Further, oral administration of *C. minuta*, isolated from NingXiang (NX) pigs, to ICR pregnant mice confirmed its maternal transmission potential. These results confirm the effectiveness of vertical transmission mechanisms as predicted by MOMTM and affirm the model's accuracy in forecasting maternal–offspring microbial dynamics. Consequently, MOMTM establishes a robust framework for elucidating complex microbial transmission mechanisms, thereby enhancing our ability to design effective microbial interventions and therapeutic strategies.

## 2 Results

### 2.1 Distinct Developmental Trajectories and Maternal Influence on Gut Microbiota in Chinese Indigenous and Commercial Pigs

**Figure 1:**
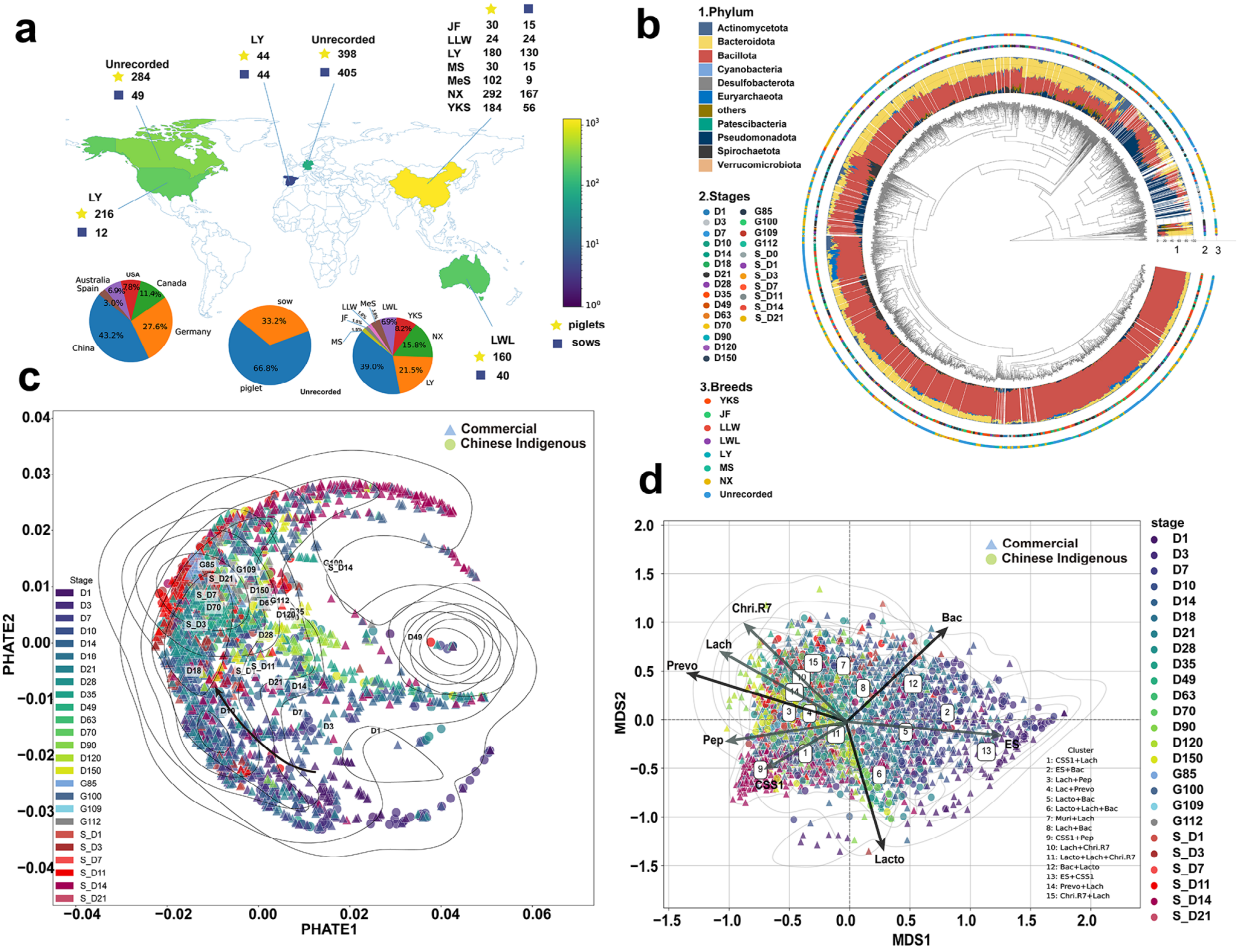
Microbial composition, diversity, and niche differentiation of Chinese indigenous and commercial pig breeds across developmental stages. a) Global distribution of pig samples across different breeds and locations. Sample distribution and proportions of fecal microbial samples collected from various pig breeds throughout the study. A total of 2910 fecal microbiome datasets were compiled, encompassing Chinese indigenous pigs (Ningxiang, Mashen, Meishan) and commercial pigs (Yorkshire, Landrace  ×  Large White, Large White  ×  Landrace). Samples were collected at multiple developmental stages ranging from birth to 150 days. b) Phylogenetic analysis of microbial composition at different developmental stages and across pig breeds. The circular phylogenetic tree illustrates the microbial community composition at the phylum level, showing the distribution of microbial taxa across different developmental stages and pig breeds. The figure consists of three annotated layers: phylum composition (inner ring): the different colors in the innermost layer represent the relative abundance of bacterial phyla including *Actinomycetota* (blue), *Bacteroidota* (yellow), *Bacillota* (red), *Pseudomonadota* (deep blue), and others; developmental stages (middle ring): the middle ring annotates the stages of development; each developmental stage is represented by a unique color, illustrating how the microbial community changes over time; pig breeds (outer ring): the outermost layer shows the different pig breeds, with each breed represented by a distinct color. Breeds include YKS (orange), JF (green), LLW (deep orange), LWL (purple), LY (cyan), MS (green), NX (yellow), and “Unrecorded” (blue). c) Multiscale potential of heat diffusion for affinity‐based transition embedding (PHATE) analysis of fecal microbiota of sows and piglets. A multiscale PHATE plot visualizing niche differentiation and developmental trajectories of piglet fecal microbiota. Each point represents a fecal sample colored by different developmental stages. Triangular points (▲) represent commercial pig breeds. Circular points (●) represent Chinese indigenous pig breeds. d) Enterotype shifts in piglets from Chinese indigenous and commercial pigs before and after weaning. Specifically, Chri.R7 denotes the *Christensenellaceae R‐7* group; CSS1 refers to *Clostridium sensu stricto 1*; Lach stands for *Lachnospiraceae*; ES represents *Escherichia–Shigella*; Bac indicates *Bacteroides*; Prevo signifies *Prevotella*; and Lacto corresponds to *Lactobacillus*.

To explore the influence of genetic background, rearing environment, and dietary composition on the developmental trajectories of the pig microbiome, we collected 892 fecal samples from sow–piglet pairs and supplemented this with 2018 publicly available samples from global databases, resulting in a total of 2910 samples for analysis (**Figure** **1**a). We categorized the pig samples into two groups: Chinese indigenous pigs and commercial pigs, which revealed both breed types were predominantly colonized by *Pseudomonadota*, *Bacillota*, and *Bacteroidota* during the lactation period. Postweaning, both groups exhibited significant declines in *Pseudomonadota* and *Actinomycetota* abundance. During development, all pigs underwent rapid microbial succession, ultimately establishing stable *Bacillota*‐ and *Bacteroidota*‐dominant communities (Figure S1a, Supporting Information). Notably, indigenous breeds maintained lower *Bacillota* levels during early developmental stages compared to exotic breed. Postweaning, exotic breeds showed higher abundances of *Pseudomonadota*, *Actinomycetota*, and *Euryarchaeota*, along with lower *Bacteroidota* levels (Figure 1b and Figure S1a (Supporting Information)). These findings demonstrate that while Chinese indigenous and commercial pig breeds share similar overall microbial developmental trajectories, they exhibit distinct compositional differences in specific bacterial phyla distributions.

To explore niche differentiation between Chinese indigenous and commercial pigs across developmental stages, we employed multiscale potential of heat diffusion for affinity‐based transition embedding (PHATE), an unsupervised dimensionality reduction method ideal for visualizing and clustering complex, noisy datasets. PHATE analysis revealed that piglet fecal microbiota transitioned from “valleys” to “hills” along contour lines, indicating significant microbial community changes. Although genetic and dietary differences influenced microbiota during lactation, niche disparities diminished as the gut microbiota matured, though notable distinctions remained between sow and mature piglet niches (Figure 1c). Analysis of the Shannon index revealed statistically significant differences between the two pig groups at 3, 7, and 28 days of age, with commercial breeds demonstrating higher average Shannon indices than Chinese indigenous breeds. Regarding fecal microbial richness, no significant differences were observed between indigenous and exotic breeds during the lactation period. However, Chinese indigenous breeds showed significantly higher richness indices than commercial breeds at 28, 90, and 150 days of age (Figure S1b,c, Supporting Information). Despite initially lower prevalence of maternal microbiota, indigenous pigs experienced a more rapid increase during lactation (Figure S1d, Supporting Information). FEAST analysis also indicated higher maternal microbial contributions in indigenous piglets on days 3 and 21 (Figure S1e, Supporting Information). The Bray–Curtis dissimilarity index showed a decreasing trend in both groups. Notably, Chinese indigenous piglets demonstrated significantly higher maternal microbial similarity at 7, 21, 28, 90, and 150 days of age compared to commercial breeds (Figure S1f, Supporting Information).

We further employed Dirichlet multinomial mixtures (DMM) modeling to classify fecal microbiota profiles of all samples, partitioning the dataset into 15 distinct clusters, which were classified into three predominant enterotypes: *Bacteroides* (Bac), *Prevotella* (Prevo), and *Lactobacillus* (Lacto) (Figure 1d). During the early developmental stages, the microbiota was predominantly composed of the  *Escherichia*–*Shigella*, followed by progressive transitions toward *Bacteroides*‐ and *Prevotella*‐dominated communities.

Comparative analysis of enterotype‐specific alpha diversity between Chinese indigenous and commercial pig breeds (with values increasing left to right) revealed that clusters 10 and 15 (driven by unclassified *Lachnospiraceae* and the *Christensenellaceae R‐7* group), exhibited the highest Shannon diversity and richness (Figure S1g,h, Supporting Information). Indigenous pigs showed higher Shannon diversity in *Clostridium sensu stricto 1* and *Muribaculaceae*‐dominated enterotypes, whereas cluster 14, dominated by *Prevotella*, comprised only commercial pig samples. Sankey diagrams (Figure S2a, Supporting Information) and scatter plots (Figure 1d) showed that indigenous pigs (circles) primarily differentiated into *Lactobacillus*‐dominated enterotypes, whereas commercial pigs (triangles) frequently transitioned into *Prevotella*‐dominated enterotypes. Sow–piglet enterotype correlations (Figure S2b, Supporting Information) showed that in commercial pigs, sow enterotypes 7 and 10 were strongly associated with piglet enterotypes 2, 12, 14, and 7. Indigenous pigs frequently paired sow enterotypes 3 and 15 with specific piglet enterotypes. Chi‐square tests and Cramér's *V* demonstrated a stronger sow influence on piglet enterotypes in commercial breeds (*V* = 0.3168 vs 0.2792). Chinese indigenous and commercial pigs show distinct gut microbiota developmental trajectories influenced by maternal contributions, diet, and genetics. Indigenous pigs exhibit faster microbial succession during lactation and distinct microbial community structures compared to commercial breeds, highlighting breed‐specific maternal effects on early microbiota establishment and maturation.

### 2.2 Maternal–Offspring Microbial Transmission Analysis Using an Attention‐Based LSTM Model

**Figure 2:**
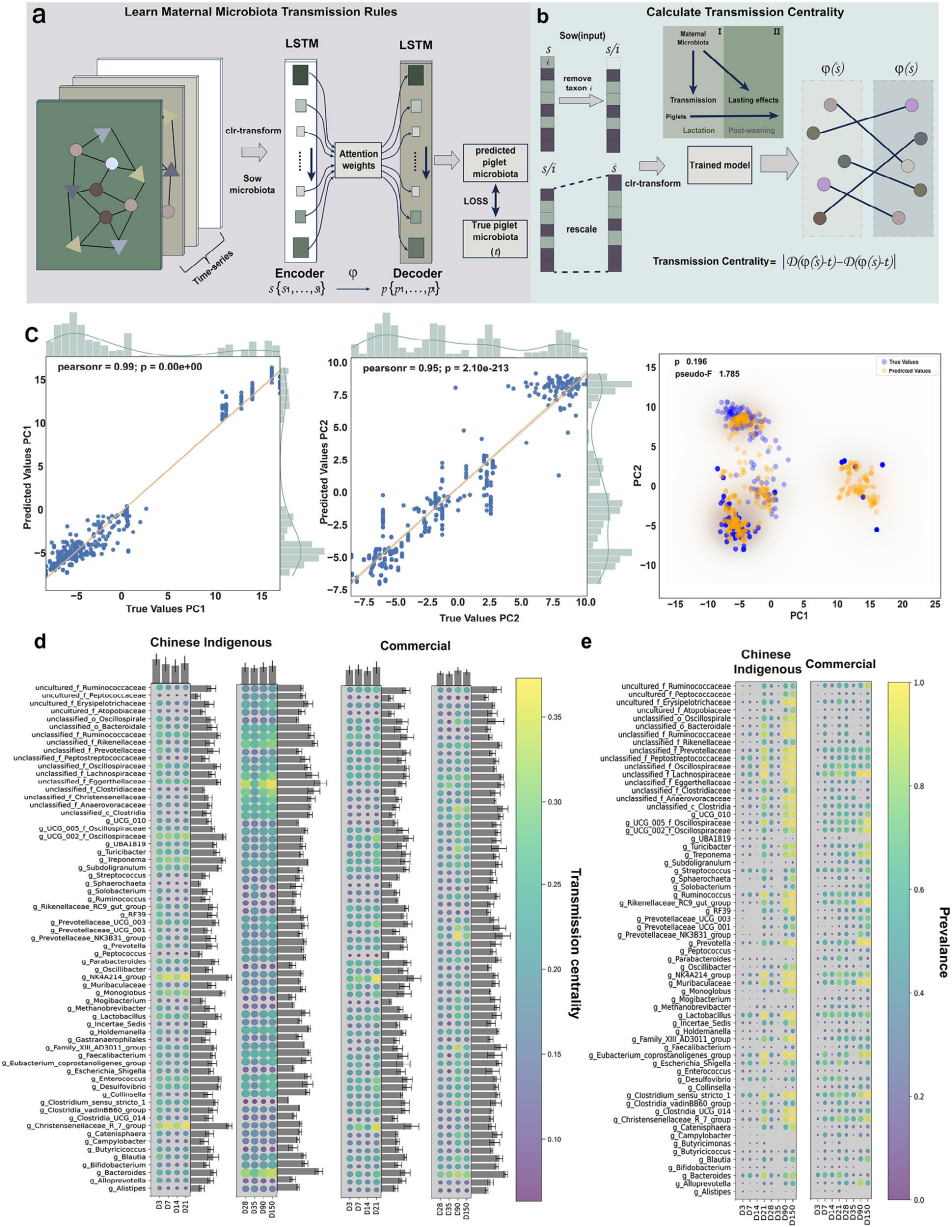
Maternal–offspring microbial transmission analysis using an attention‐based LSTM model. Schematic of the maternal–offspring microbial transmission model (MOMTM).a) Diagram illustrating the encoder–decoder architecture of the MOMTM. The encoder processes time‐series maternal microbiota data to capture temporal dynamics, and the decoder predicts piglet microbiota compositions at different time points. An attention mechanism highlights key microbial taxa influencing transmission. b) Ablation study results highlighting key microbial taxa. Each taxon was individually removed from maternal data, and the change in piglet microbiota predictions (ΔD*i*) was calculated. Higher transmission centrality (TC) values indicate greater influence on microbial transmission. c) Principal component analysis (PCA) comparing predicted and actual piglet microbiota compositions. Scatter plots of the first two principal components (PC1 and PC2) from PCA, comparing predicted and actual microbiota compositions of piglets, showing correlation coefficients. Pearson correlation coefficients (*r*) for PC1 (*r*  =  0.99) and PC2 (*r*  =  0.95) in testing set using PERMANOVA test demonstrate strong model performance. d) Transmission centrality of key taxa at different stages in Chinese indigenous and commercial pigs. Bar plots (right and top) show mean ± SEM of TC values for key taxa. e) Prevalence of maternal positive bacteria in piglets at different lactation and postweaning stages.

To further investigate the significance of different microbial taxa in maternal transmission, we developed MOMTM to characterize the dynamics of mother–offspring microbiome transmission. Our approach comprises two main phases. In the first phase (**Figure** **2**a), MOMTM is trained on longitudinal microbiome data to implicitly learn microbial transmission patterns from sows to piglets. The encoder captures temporal and compositional features of maternal microbiota, while the decoder forecasts offspring microbiota profiles at subsequent stages. To identify pivotal maternal microbes influencing piglet gut microbiota, we performed an ablation study using the trained model (Figure 2b). Each microbial taxon *i* was systematically removed from maternal data, and transmission centrality (TC) was assessed by calculating the Euclidean distance (Δ*D*(*i*)) between original and modified predictions.

To enhance model stability, lactation and postweaning data were divided into four stages. The model was trained for 150 epochs, with loss converging to ≈40, mean squared error (MSE) decreasing to ≈2, and mean absolute error (MAE) reducing to ≈1.1. The coefficient of determination (*R* ^2^) plateaued around 0.48 (Figure S3a, Supporting Information). Tenfold cross‐validation yielded MSE between 0.7 and 1.28, MAE between 0.32 and 0.53, and *R* ^2^ from 0.33 to 0.48 (Figure S3b, Supporting Information). Principal component analysis (PCA) of predicted versus actual values revealed Pearson correlations of 0.99 for PC1 and 0.95 for PC2 (Figure 2c).

During lactation, TC indicates maternal microbes facilitate microbial transmission and community establishment in piglets, while postweaning TC suggests long‐term influence. Indigenous pigs exhibit higher TC during days 1–3 and 14–21 of lactation, whereas commercial pigs peak between days 3–7 and 14–21, indicating two critical windows with an earlier peak in indigenous breeds. This corresponds with FEAST results (Figure 1d), showing significantly higher maternal microbes in indigenous piglets at days 3 and 21. Our ablation study highlights the *Christensenellaceae R‐7* group as having high TC in both pig breeds, with indigenous pigs showing high centrality at days 3–7 and 14–21, and commercial pigs only at days 14–21 (Figure 2d).

Postweaning, influential maternal microbes in indigenous pigs included unclassified *Eggerthellaceae*, *Bacteroides*, unclassified *Rikenellaceae*, and *Lachnospiraceae*. In commercial pigs, the influential taxa were *Prevotella*, *Bacteroides*, and the *Prevotellaceae NK3B31* group (Figure 2d). Maternal positive bacteria prevalence was higher in indigenous pigs at days 14–21, 35–90, and 90–150 (Figure 2e). During lactation, the correlation between TC and maternal bacteria was stronger in indigenous pigs, while commercial pigs showed higher correlations postweaning (Figure S3c, Supporting Information). Building on the observed differences in gut microbiota development between pig breeds, our analysis of maternal–offspring microbial transmission reveals that maternal–offspring microbial transmission is shaped by critical windows during lactation, with *Christensenellaceae R‐7* group identified as a key maternal taxon influencing piglet gut microbiota, highlighting breed‐specific dynamics.

### 2.3 Targeted Maternal Microbial Intervention Alters Offspring Enterotypes and Microbial Functions

Our MOMTM model identified the *Christensenellaceae R‐7* group as having high TC during lactation (Figure 2d), and enterotypes dominated by *Christensenellaceae R‐7* group in the offspring displayed elevated alpha diversity (Figure S1g,h, Supporting Information). We therefore hypothesized that targeted modulation of maternal *Christensenellaceae R‐7* group could enhance its transmission to the offspring.

**Figure 3:**
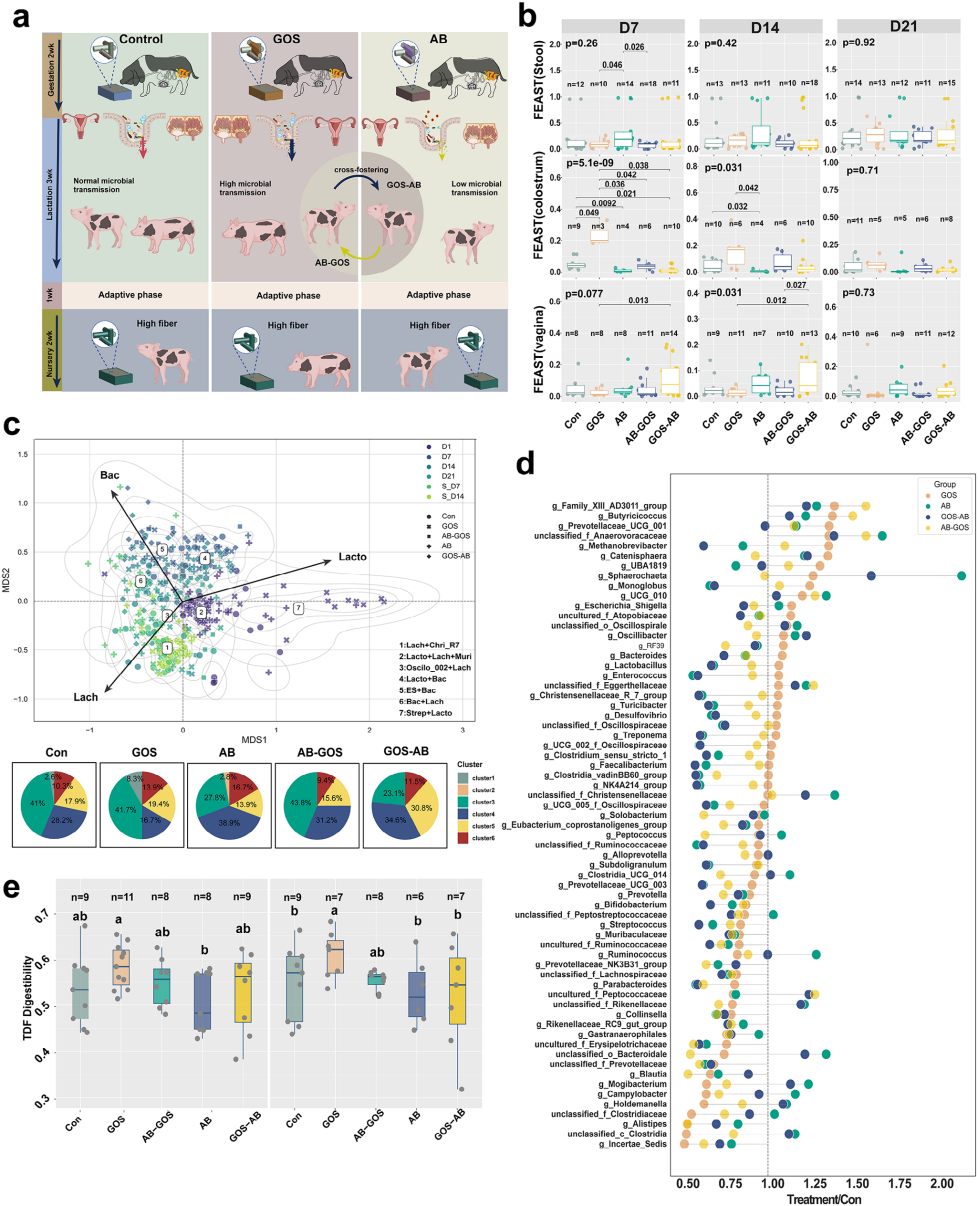
Maternal microbial intervention alters offspring microbiota and fiber digestibility. a) Experimental design of maternal interventions and cross‐fostering. Schematic illustrating the study groups—Control, galacto‐oligosaccharide supplementation (GOS), cross‐fostered from GOS to AB at birth (GOS–AB), cross‐fostered from AB to GOS at birth (AB–GOS), and antibiotics (AB)—including timing of interventions, cross‐fostering procedures, and subsequent high‐fiber diet feeding. b) FEAST analysis estimating the contributions of maternal colostrum, vaginal, and fecal microbiota to piglet fecal microbiota at days 7, 14, and 21. c) Enterotype distribution in maternal and piglet samples and proportional distribution of enterotypes among experimental treatment groups in piglets (pie chart representation). d) TC analysis using the MOMTM model across five treatment groups, normalized by control. e) Total dietary fiber (TDF) digestibility in piglets at days 35 (left) and 45 (right).

To determine which oligosaccharide most effectively promotes the transmission of *Christensenellaceae R‐7* group, we employed random forest analysis to evaluate the regulatory effects of four oligosaccharides—xylo‐ologosaccharides (XOS), isomalto‐oligosaccharides (IMO), arabinose‐based oligosaccharides (ASO), and GOS—on *Christensenellaceae R‐7* group. The analysis revealed that GOS possessed the greatest potency in modulating *Christensenellaceae R‐7* group populations (Figure S4a and Table S1, Supporting Information), which suggested that GOS‐mediated modulation may further enhance the vertical transmission of the *Christensenellaceae R‐7* group from mother to offspring. Subsequently, the experimental design involved three treatment groups for sows: 1) control group (standard diet), 2) GOS‐supplemented group (diet with GOS), and 3) antibiotic‐treated group (receiving antibiotic treatment, AB). Following birth, piglets were cross‐fostered between GOS and AB groups to evaluate the effects of maternal microbiota. One weeks of weaning, all piglets were weaned onto a high‐fiber diet to assess developmental impacts on gut microbiota (**Figure** **3**a). Importantly, Tables S4–S6 (Supporting Information) indicate minimal effects of maternal interventions on reproductive parameters, milk composition, and piglet initial body weights. Specifically, maternal GOS supplementation improved piglet average daily gain (Table S4, Supporting Information) and significantly increased the healthy litter size (Table S5, Supporting Information), while other parameters, including litter size, IUGR rate, milk composition, and piglet birth weights, remained unaffected (Tables S4–S6, Supporting Information). FEAST analysis demonstrated that sows treated with GOS exhibited significantly enhanced colostrum contributions to piglet microbiota on days 7 and 14, whereas AB‐treated sows showed the opposite effect. By day 21, these differences were no longer significant (Figure 3b).

Using the DMM model, we classified microbiota of both treated sows and their piglets over time. In sows’ colostrum, cluster 2 (92.2%, dominated by *Lactobacillus*, *Lachnospiraceae*, and *Muribaculaceae*) and cluster 5 (7.8%, dominated by *Escherichia–Shigella*) prevailed. GOS‐treated and AB‐treated sows showed an increase in cluster 2 and a decrease in cluster 5. In vaginal swabs from sows, cluster 2 (40.9%), cluster 5 (9.1%), and cluster 7 (50.0%, by *Streptococcus* and *Lactobacillus*) were observed AB treatment promoted cluster 2, GOS enhanced cluster 7, and cluster 5 was exclusive to AB‐treated sows (Figure S4b, Supporting Information).

Microbiota analysis of piglets from GOS‐ and AB‐treated sows revealed that maternal GOS supplementation preferentially increased cluster 1 (*Lachnospiraceae* and *Christensenellaceae R‐7* group), whereas maternal AB treatment promoted clusters 2 and 4 (*Lactobacillus* and *Bacteroides*) while suppressing cluster 3 (*Oscillospiraceae*) during the lactation period (Figure 3c). Cross‐fostered piglets mirrored their new groups’ microbiota, whereas postbirth GOS had no effect, indicating gestational or delivery‐related antibiotics affect lactation‐stage microbiota.^[^ ^27^ ^]^ Prenatal antibiotics and GOS significantly altered vaginal microbiota of sows, with antibiotics increasing *Deferribacterota* and *Gemmatimonadota*, and GOS enriching *Bacillota* (Figure S4c, Supporting Information).

Figure S4d (Supporting Information) shows that from days 7 to 21 of lactation, there were no significant differences in the gut microbiota between piglets from nonkin and kin sows. Separately, significant differences were observed across all five groups when comparing sow vaginal microbiota with piglet fecal microbiota at day 7, sow fecal microbiota at day 14 with piglet fecal microbiota at day 21. Moreover, the Bray–Curtis dissimilarity gradually decreased, with the GOS group showing the lowest dissimilarity at day 21 (Figure S4e, Supporting Information).

In piglets, at day 7, there was a marginal trend in overall Shannon diversity (*p* = 0.052). Richness differed significantly (*p* = 0.013), with the GOS group showing higher richness than the AB–GOS group (*p* = 0.023). By day 14, the GOS group exhibited higher diversity than both the AB (*p* = 0.0045) and GOS–AB (*p* = 0.0023) groups, while the AB group had lower diversity compared to the Control (*p* = 0.01) and AB–GOS (*p* = 0.0031) groups. Richness remained significantly different (*p* = 0.013), with the AB group showing lower richness than the Control (*p* = 0.0027), GOS (*p* = 0.0012), and AB–GOS (*p* = 0.012) groups. By day 21, both Shannon diversity (*p* = 0.49) and richness (*p* = 0.82) converged among the treatment groups (Figure S4f, Supporting Information).

To investigate how different maternal microbial interventions affect short‐chain fatty acid (SCFA) profiles in piglet colon and cecum, we measured SCFA levels in colonic contents at 21 days of age. In the colon (Figure S4g, Supporting Information), the GOS, AB–GOS, and GOS–AB groups showed significantly higher propionate levels compared to the control (*p* = 0.007, 0.00058, 0.038, respectively). Additionally, the AB and AB–GOS groups had significantly higher acetate levels than the control (*p* = 0.011 and 0.00058, respectively). By contrast, no significant differences in SCFA levels were observed among the groups in the cecum (Figure S4h, Supporting Information). Mantel analysis identified significant correlations between SCFA profiles and colon microbiota in piglets’ colon. *Unclassified_Oscillospiraceae* was positively correlated with acetate and propionate, *Lachnospiraceae* with isobutyrate and butyrate, *Ruminococcaceae* with acetate and propionate, and *Prevotella* showed strong positive correlations with acetate, propionate, and butyrate (*p* < 0.01) (Figure S4i, Supporting Information). Network analysis of piglet colon microbiota revealed five interaction clusters, the largest involving *Christensenellaceae R‐7* group and *NK4A214*, with *Christensenellaceae R‐7* group exhibiting high centrality metrics, highlighting its pivotal network role (Figure S4j and Table S7, Supporting Information). Applying the MOMTM model to five groups of piglets and normalizing TC against control revealed that in piglets from GOS treatment sows, key taxa such as the *Christensenellaceae R‐7* group had significantly higher TC, indicating enhanced maternal transmission efficiency. By contrast, the AB exhibited lower TC values of *Christensenellaceae R‐7* group (Figure 3d).

This study further evaluated the impact of maternal microbial interventions on piglet dietary fiber utilization by measuring total dietary fiber (TDF) digestibility at 35 and 45 days of age. At 35 days of age, significant differences in TDF digestibility were observed among groups. Piglets from sows fed GOS exhibited markedly higher TDF digestibility compared to the AB group. Following a 10 days adaptation to a high‐fiber diet (day 45), TDF digestibility increased in all groups, while piglets from the GOS‐treated sows maintained significantly higher TDF digestibility than those in the Con, AB, and GOS–AB groups (Figure 3e). Further in‐depth analysis of carbohydrate‐active enzyme (CAZyme) families (Figure S4k, Supporting Information) revealed 34 families with significant differential abundance. Notably, GT106, GT10, GT35, GT18, CE11, CE9, and CBM54 were enriched in piglets from GOS‐treated sows, whereas GH171, CE1, GT22, GH65, GT26, GH170, GH1, and GH13 showed increased abundance in piglets from the antibiotic‐treated sows. The gut microbiota across treatment groups exhibited significant divergence in KEGG pathways (Figure S4l, Supporting Information), with the AB and GOS–AB groups demonstrating markedly higher activity in the galactose metabolism, cyanoamino acid metabolism, nicotinate and nicotinamide metabolism, and oxidative phosphorylation compared to GOS and AB–GOS groups. Notably, microbial metabolism in diverse environment and the phosphotransferase system were substantially enriched in AB and GOS–AB groups. Glycine, serine, and threonine metabolism predominated in AB and GOS–AB groups, while arginine and proline metabolism showed greater abundance in the GOS group. Conversely, microbial structural pathways such as peptidoglycan biosynthesis and bacterial secretion systems were significantly attenuated in AB and GOS–AB groups. Vitamin metabolic pathways, particularly biotin metabolism and sulfur metabolism, were significantly elevated in GOS and AB–GOS groups relative to other treatments. Colonic crypt depth remained similar across groups (Figure S4m, Supporting Information).

### 2.4 Functional Enrichment and Colonization Strategies of *Christensenella* Species across Developmental Stages

**Figure 4:**
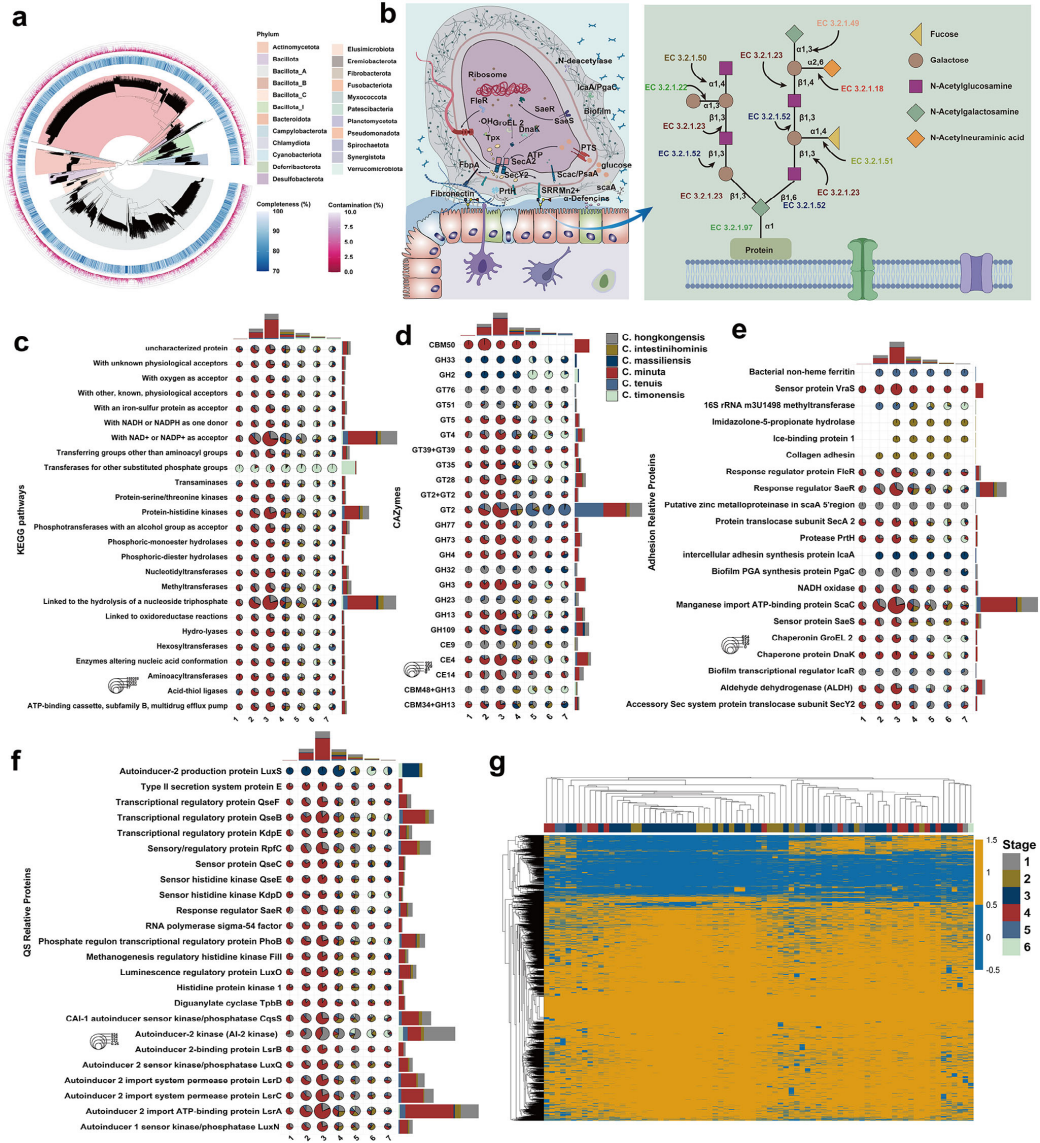
Functional enrichment and colonization strategies of *Christensenella* species across developmental stages. a) Phylogenetic tree of MAGs. Phylogenetic tree constructed from 6775 medium‐quality metagenome‐assembled genomes (MAGs) (completeness > 70%, contamination < 10%). b) Schematic of bacterial adhesion and mucin‐degrading pathways. c) KEGG functional enrichment of *Christensenella* species across developmental stages (weeks 1–7). d) Carbohydrate‐active enzymes (CAZyme) profiles of *Christensenella* species. e) Adhesion‐related genes and f) quorum sensing genes in different *Christensenella* species across developmental stages (weeks 1–7). The bar plots at the top represent the overall distribution of *Christensenella* species from weeks 1 to 7. g) Gene family detection of *C. minuta* using PanPhlAn across 415 samples.

Based on the MOMTM model's identification of the *Christensenellaceae R‐7* group as having the highest TC during lactation (Figure 2d), we next sought to characterize its functional repertoire across developmental stages. To this end, we reconstructed 6775 medium‐quality metagenome‐assembled genomes (MAGs; ≥70% completeness, ≤10% contamination) from 451 piglet fecal metagenomes. We then computed average nucleotide identity (ANI) between each MAG and six reference genomes of *Christensenella* species. MAGs exhibiting ANI > 95% to a given reference were designated as that species’ representative for all downstream functional and pathway enrichment analyses (**Figure** **4**a). KEGG enrichment analysis indicated that the top 25 pathways show a progressive increase during the first three weeks of lactation, followed by a steady decrease postweaning. Concurrently, *C. minuta* demonstrated stronger functional enrichment within this window, while *Christensenella timonensis* displayed more pronounced enrichment after weaning. Notably, these top 25 enriched functions predominantly involve NADP⁺ as an acceptor, protein–histidine kinases, and processes linked to nucleoside triphosphate hydrolysis (Figure 4c). CAZyme profiles varied significantly among *Christensenella* species, indicating niche differentiation in carbohydrate utilization, with *C. minuta* possessing a broader range of enzyme functions (Figure 4d). Microbial colonization in the mammalian gut mainly involves bacteria adhering to intestinal mucus or epithelial cells through adhesion mechanisms.^[^ ^28^ ^]^ Adhesion enables bacteria to withstand intestinal movements, supporting growth and reproduction, and promotes biofilm formation, enhancing survival against immune defenses.^[^ ^29^, ^30^ ^]^ Several *Christensenella* species exhibit distinct adhesion‐related strategies. Specifically, *Christensenella intestinibominis* employs imidazolone‐5‐propionate hydrolase, ice‐binding protein 1, and collagen adhesin to facilitate adhesion. By contrast, *Christensenella massiliensis* relies predominantly on the intercellular adhesin synthesis protein IcaA. *Christensenella tenuis* utilizes bacterial nonheme ferritin, while *Christensenella hongkongensis* is characterized by biofilm PGA synthesis protein PgaC and a putative zinc metalloproteinase. Notably, *C. minuta* demonstrates enhanced functionality across multiple adhesion mechanisms, including sensor protein *VraS*, manganese import ATP‐binding protein *ScaC*, and chaperonin *GroEL 2*. These variations underscore the specialized roles each *Christensenella* species plays in microbial adhesion and interaction within their respective environments (Figure 4e). Quorum sensing analysis revealed that *Christensenella* species exhibit distinct temporal patterns of signaling activity in piglets. Specifically, *C. minuta* and *C. hongkongensis* demonstrated robust quorum sensing mechanisms during the lactation period. By contrast, postweaning stages were predominantly characterized by enhanced quorum sensing activity in *C. intestinibominis* and *C. timonensis*. These observations suggest a dynamic shift in quorum sensing strategies among *Christensenella* species, potentially reflecting their adaptive roles in the host's microbiome during different developmental phases (Figure 4f). To accurately characterize the distribution of *C. minuta* across developmental stages, we constructed a pangenome database using pangenome‐based phylogenomic analysis (PanPhlAn) and identified gene families present at each stage. Among 451 samples, *C. minuta* was detected in 79 samples, with the highest prevalence (49.4%) observed during the third week of lactation (Figure 4g).

To elucidate functional distinctions between high and low prevalence maternal bins, we reconstructed 2202 medium‐quality MAGs (≥70% completeness, ≤10% contamination) from 231 fecal samples collected from genetically related sows and piglets (31 sows and the corresponding 200 piglets) across developmental stages sourced from publicly available databases (Figure S5a, Supporting Information). During lactation, the top 10% most prevalent bins exhibited significantly greater enrichment in key functional pathways compared to the bottom 10%. These differences diminished over the first three days before resurging thereafter. Following weaning, the functional enrichment of the top 10% prevalent bins declined relative to the lactation period (Figure S5b, Supporting Information). Colonic mucus is predominantly composed of mucin 2 (*Muc2*), a glycoprotein containing up to 80% glycans by mass and over 100 O‐linked glycan structures on serine or threonine residues. Interactions between commensal microbiota and host mucins are essential for gut colonization, with microbiota‐mediated utilization of mucin glycans affecting mucus properties and gut health.^[^ ^31^ ^]^ The top 10% bins also showed higher CAZyme enrichment during lactation, with the largest functional differences on day one, which then declined and rose again. GT2, GT4, and GH2 were the most enriched enzymes (Figure S5c, Supporting Information). Adhesion‐related gene annotation (Figure S5d, Supporting Information) showed that a significant enrichment of genes encoding biofilm poly‐β‐1,6‐*N*‐acetylglucosamine (GlcNAc) synthase during lactation, consistent with an enhanced capacity for biofilm formation. Poly‐beta‐1,6‐*N*‐acetyl‐d‐glucosamine *N*‐deacetylase creates a cohesive extracellular matrix, enabling strong intercellular connections via electrostatic interactions with bacterial cell surface teichoic acids.^[^ ^32^ ^]^ Subsequently, the response regulator *SaeR* and manganese import ATP‐binding protein *ScaC* were expressed. *SaeRS* regulates over 20 virulence factors, including toxins and enzymes, increasing susceptibility to host immune responses.^[^ ^33^ ^]^ Analysis of quorum‐sensing (QS)‐related genes annotation revealed that the top 10% most prevalent genomic bins during early lactation exhibited significantly higher functional abundance, which subsequently declined in later lactation stages. Adhesion‐related gene annotation (Figure S5e, Supporting Information) revealed that genes involved in quorum‐sensing autoinducer pathways were the most abundant, notably *luxS* (autoinducer‐2 synthase), *ai‐2 kinase*, and *lsrA* (autoinducer‐2 import ATP‐binding protein). This was followed by elevated abundance of transcriptional regulator genes such as *qseF*, *qseB*, *phoB*, and *kdpE*, while sensor kinase genes *VraS* and *qseC* were moderately abundant but still significantly represented (Figure S5e, Supporting Information).

To investigate how colonizing bacteria utilize host mucins, we extracted enzyme commission (EC) numbers corresponding to mucin‐degrading glycosidic bonds (Figure 4b). Across all stages, the pathway EC 3.2.1.23, responsible for degrading β‐1,3 and β‐1,4 linkages, was the most abundant, followed by EC 3.2.1.52, targeting β‐1,3 bonds (Figure S5f, Supporting Information). Analysis of the average relative abundance and prevalence of *Christensenella* species in offspring revealed that *C. minuta* consistently maintained a high relative abundance throughout the lactation period. Notably, *C. minuta* exhibited a dynamic trajectory, initially increasing, followed by a subsequent decline, and then rising again before decreasing postweaning (Figure S5g, Supporting Information). Mucin degradation pathways were significantly more extensive in *C. minuta*, primarily involving glycoside hydrolases GH2, GH20, GH27, and GH3. Notably, GH2 was present across all five *Christensenella* species examined, whereas *C. massiliensis* lacked any mucin‐degradation‐related enzymes genes (Figure S5h, Supporting Information).

### 2.5 Maternal Supplementation with *C. minuta* Alters Offspring Gut Microbiota and Metabolism

**Figure 5:**
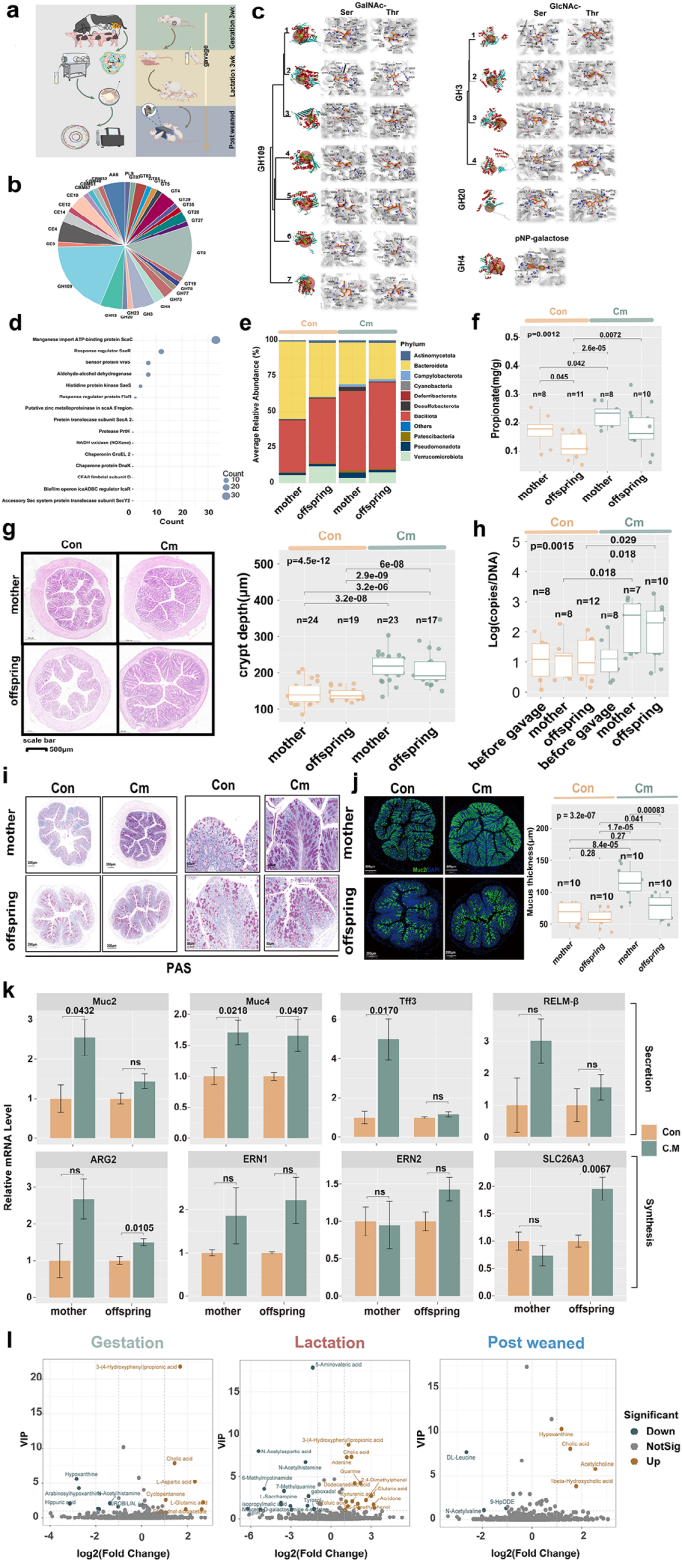
Functional role of *C. minuta* in offspring gut microbiota and metabolism. a) The work flow showing the isolation of *C. minuta* from NX pig feces, followed by administration of *C. minuta* to pregnant mice. b) Pie chart of the functional categories of CAZymes encoded in the *C. minuta* genome. c) Molecular docking of mucin‐degrading enzymes with mucin. The docking of *C. minuta* mucin‐degrading enzymes—GH109 with GalNAc‐Ser/Thr, GH20 and GH3 with GlcNAc‐Ser/Thr, and GH4 with pNP‐galactose. Docking simulations were performed using AutoDock Vina with grid box dimensions of 30  ×  30  ×  30 Å. The blue dotted lines indicate the presence of interactions between specific active sites of the enzymes and the substrates. d) Adhesion‐Related genes of *C. minuta*. e) The relative abundances of major bacterial phyla in the fecal microbiota of dams and offspring in control and *C. minuta*‐treated groups. f) Concentrations of propionate in colon chyme of dams and offspring following *C. minuta* treatment. g) Histological analysis of colon crypt depth. Scale bars represent 500 µm. h) Absolute quantification of *C. minuta* 16S rDNA gene per gram of feces in dams and offspring, determined by quantitative real‐time PCR (qPCR). i) PAS staining of maternal and offspring colons at the end of lactation period. j) *Muc2* immunofluorescence, and k) expression of genes related to mucin synthesis and secretion (*n* = 5). l) Volcano plots displaying significantly altered metabolites in colonic chyme of dams during pregnancy and lactation, and in offspring postweaned, between control and *C. minuta*‐treated groups (VIP > 1, |log₂FC| > 1). Metabolites were annotated using UHPLC–MS and analyzed with Compound Discoverer 3.3. *N*_mother_ (Control) = 9, *N*_offspring_ (Control) = 11, *N*_mother_ (*C. minuta*) = 8, *N*_offspring_ (*C. minuta*) = 10. Multiple group comparisons were conducted using the Kruskal–Wallis test, followed by Dunn's post‐hoc test with Benjamini–Hochberg correction for multiple comparisons.

To further elucidate the role of *C. minuta* in maternal–offspring microbial transmission, we isolated *C. minuta* from fecal samples of NX pigs and administered it via oral gavage to pregnant ICR mice (**Figure** **5**a). Its phylogenetic relationship to other *Christensenellaceae* strains reported in NCBI is depicted in Figure S6a (Supporting Information). Transmission electron microscopy (TEM) confirmed its characteristic fusiform shape with tapered ends, typical of the *Christensenellaceae* family (Figure S6b, Supporting Information). Whole‐genome sequencing revealed a primary focus on carbohydrate metabolism (Figure S6c, Supporting Information). KEGG pathway analysis revealed that ABC transporters were the most significantly enriched pathway, followed by biosynthesis of amino acids and carbon metabolism (Figure S6d, Supporting Information). Additionally, genomic characterization of *C. minuta* identified a repertoire of functionally annotated genes associated with probiotic traits (Table S8, Supporting Information), comprising 22 acid resistance genes, 1 bile salt tolerance gene, 2 adhesion‐related genes, 4 antioxidant genes, 1 riboflavin biosynthesis gene, 25 propionate biosynthesis genes, 6 heat shock resistance genes, 2 cold stress adaptation genes, and 1 organic acid synthesis gene. CAZyme analysis showed GH109 was the most abundant enzyme family (20%), followed by GT2 (12.8%), with GH3, GH4, and GH20—associated with mucin degradation—present at 5.7%, 2.8%, and 1.4%, respectively (Figure 5b). Phylogenetic analysis of GH109 enzymes from *C. minuta* and NCBI sequences identified two closely related pairs among three groups, while one enzyme exhibited more distant homology (Figure S6e, Supporting Information). We performed molecular docking analyses to elucidate the interactions between *C. minuta* mucin‐degrading enzymes GH109, GH20, GH3, and GH4 with their respective substrates: GH109 with GalNAc‐Ser/Thr, GH20 and GH3 with GlcNAc‐Ser/Thr, and GH4 with pNP‐galactose. Each enzyme was evaluated using up to ten models, with top‐ranked models indicating the highest binding affinities (Tables S9–S11, Supporting Information). GH109 variants (GH109_1 to GH109_7) exhibited binding energies ranging from −4.8 to −7.7 kcal mol⁻¹, with GH109_2 showing the strongest affinity for GalNAc‐Ser/Thr at −7.7 kcal mol⁻¹. GH3 enzymes demonstrated significant interactions with GlcNAc‐Ser/Thr, notably GH3_3 with a binding energy of −7.5 kcal mol⁻¹. GH20 also displayed strong affinities for GlcNAc‐Ser/Thr, with top binding energies of −6.7 kcal mol⁻¹. GH4, tested with pNP‐galactose, achieved a binding energy of −6.3 kcal mol⁻¹. These results indicate that GH109 and GH3 enzymes have specific substrate preferences, reflecting their roles in mucin O‐glycan degradation. Detailed 3D docking models illustrating these enzyme–substrate interactions are presented in Figure 5c.

To assess the gut adhesion capabilities of *C. minuta* from NX pigs, we annotated its genome using an adhesin database. The analysis revealed *ScaC* (a manganese import ATP‐binding protein) as the most abundant adhesion‐related genes (highest copy number), followed by genes encoding the response regulator *SaeR* and sensor protein *VraS* (Figure 5d). Pregnant ICR mice were orally administered *C. minuta* throughout gestation and lactation. The treated group exhibited an increased abundance of *Bacillota* and a decreased abundance of *Bacteroidota* in both dams and offspring compared to controls (Figure 5e). Nonmetric multidimensional scaling (NMDS) analysis revealed distinct clustering separation between treated and control groups (Figure S6f, Supporting Information), although no significant differences were observed in Shannon diversity or richness indices (Figure S6g, Supporting Information). Interestingly, the concentration of propionate in the colonic contents of *C. minuta*‐treated dams was significantly elevated compared to controls, a metabolic pattern that was consistently observed in their offspring (Figure 5f). KEGG pathway analysis of the *C. minuta* genome revealed the presence of multiple propionate synthesis genes (Figure S6h (Supporting Information), blue highlights indicate gene positivity; see complete gene list in Table S8 in the Supporting Information). Histological analysis showed that colonic crypt depth was significantly increased in both mothers and offspring in the *C. minuta* group compared to controls (Figure 5g). We assessed the relative abundances of key bacterial taxa, including the *Christensenellaceae R‐7* group, *NK4A214* group, and *UCG‐005* from *Oscillospiraceae*. While no significant differences were found in the *Christensenellaceae R‐7* and *NK4A214* groups, *UCG‐005* was significantly more abundant in *C. minuta*‐treated dams (Figure S6i, Supporting Information). Absolute quantification confirmed a significant increase in *C. minuta* copies in the feces of both dams and offspring postgavage (Figure 5h).

Mucin‐related parameters were analyzed in colonic tissues from dams and their offspring at late lactation. Compared to control groups, mice treated with *C. minuta* exhibited increased numbers of mucus‐producing goblet cell in the colon (Figure 5i). Concurrently, the expression level of *Muc2* protein was significantly higher in colonic tissues from *C. minuta*‐treated dams and offspring compared to controls (Figure 5j). Specifically, regarding mucin‐secretion‐related genes, maternal colons from the *C. minuta* group showed significantly elevated expression of *Muc2* (*p* = 0.0432) and Tff3 (*p* = 0.0170) compared to controls, whereas offspring displayed no significant differences between groups. The expression of the *Muc4* gene was significantly higher in both dams (*p* = 0.0218) and offspring (*p* = 0.0497) in the *C. minuta* group compared with their respective controls. Although Relm‐β expression was consistently higher in the *C. minuta* group for both dams and offspring, these differences were not statistically significant. For mucin‐synthesis‐related genes, *Arg2* expression was significantly increased in offspring from the *C. minuta* group (*p* = 0.0105), but no significant difference was observed in dams. *Ern1* and *Ern2* expression levels were elevated in the *C. minuta* group compared to controls in both dams and offspring, but these differences were not statistically significant. Additionally, *Slc26a3* expression was significantly upregulated in offspring from the *C. minuta* group (*p* = 0.0067), whereas maternal expression remained unchanged (Figure 5k).

Metabolomic profiling of colonic chyme from dams at the end of pregnancy and from dams and offspring at the end of lactation revealed significant metabolic alterations between *C. minuta* and control groups (Figure 5i). In late‐pregnancy maternal mice (Table S13, Supporting Information), a total of 14 metabolites exhibited significant changes (|log₂fold change (FC)| > 1 and variable importance in projection (VIP) > 1). Several metabolites exhibited notable changes, including increased levels of 3‐(4‐hydroxyphenyl) propionic acid, cholic acid, and l‐aspartic acid in the Cm group. Conversely, hypoxanthine, arabinosylhypoxanthine, and *N*‐acetylhistamin were significantly decreased. During late lactation (Table S14, Supporting Information),32 metabolites showed significant changes. The Cm group showed increased levels of cholic acid, adenine, guanin, and deoxycholic acid. Conversely, significant decreases were observed in *N*‐acetylaspartic acid, *N*‐acetylhistamin, and 5‐aminovaleric acid. In postweaning offspring (Table S15, Supporting Information), 7 metabolites exhibited significant alterations. Metabolites such as acetylcholine, cholic acid, and 1β‐hydroxycholic acid were markedly upregulated in the Cm group. Conversely, dl‐leucin and buprenorphine showed significant downregulation. These metabolites suggest enhanced cholinergic signaling and altered bile acid metabolism associated with *C. minuta* treatment.

## 3 Discussion

Through long‐term coevolution, gut microbiota and their hosts have formed an interdependent and dynamically regulated symbiotic system. Host genetic background, rearing management, nutritional supply, and pharmaceutical interventions can significantly shape gut microbial composition and metabolic functions.^[^ ^34^ ^]^ Delivery mode notably affects the initial functional characteristics of the offspring microbiome; for instance, infants born by cesarean section often lack functional strains (e.g., *Lactobacillus* spp.) typically acquired through the birth canal, resulting in impaired microbial capacity for SCFA synthesis.^[^ ^35^, ^36^ ^]^ Continuous maternal microbial input from shared living environments enhances environmental adaptability and functional redundancy of offspring microbiomes.^[^ ^37^, ^38^ ^]^ Indeed, gut microbiome colonization and functional development originate through maternal transmission, profoundly shaping offspring microbial metabolism and immune regulatory capabilities. By modulating maternal microbial transfer in sows, we were able to demonstrably alter the functional properties of the piglet gut microbiota (Figure 3e).

Studies have demonstrated that maternal gut microbiota constitute a core source of microbial transmission, with ≈50.7% of neonatal microbiota at birth derived from maternal gut, vaginal, oral, and skin microbiomes. Notably, maternal gut microbiota contribute up to 22.1% of these bacteria, exhibiting sustained transmission of functional genes such as SCFA synthesis enzymes.^[^ ^39^ ^]^ Our findings further demonstrate that GOS supplementation significantly enhances the maternal microbiota of milk to offspring gut microbiota compared to control (from ≈20% vs ≈5%). Moreover, piglets from GOS‐treated sows exhibited a marked increase in colonic propionate content compared to control (Figure 3b and Figure S4g (Supporting Information)).

Maternal microbiota vertically transmit metabolic functionalities, integrating into offspring microbiome to construct a host–microbial cometabolic network. Maternal metabolic reprogramming mediated via the gut–mammary axis precisely delivers microbial‐modified immunological molecules (e.g., sIgA) into breast milk.^[^ ^40^ ^]^ Specifically, sIgA neutralizes pathogens (e.g., virulent *Bacteroides fragilis*) through fimbriae‐binding or toxin‐neutralizing mechanisms,^[^ ^41^ ^]^ while simultaneously enhancing mucosal colonization by beneficial commensal bacteria (e.g., *Lactobacillus* spp.) via glycan‐dependent aggregation, thereby optimizing microbial functional equilibrium.^[^ ^42^ ^]^ Wu et al. also demonstrated that maternal GOS supplementation during late gestation enhances offspring development through microbial and immunological programming. The intervention improved piglet growth performance, elevated IgA levels in both sows and offspring, and enriched beneficial maternal microbiota that were vertically transmitted. GOS administration strengthened intestinal barrier function in piglets by upregulating tight junction protein, increasing goblet cell numbers and *Muc2* expression, and boosting fecal acetate levels.^[^ ^43^ ^]^ These findings are consistent with our observations from GOS intervention in NX sows.

Recent data‐driven research of microbial communities provides new opportunities for finding keystone species.^[^ ^17^, ^19^ ^]^ Wang et al.^[^ ^19^ ^]^ proposed a novel framework called data‐driven keystone species identification, which enables the prediction of the “keystoneness” of each species by performing thought experiments simulating the removal of each species. Wu et al.^[^ ^21^ ^]^ proposes a data‐driven method—independent of specific dynamical models—for predicting whether and how an exogenous species will colonize a complex microbial community. Top‐down framework was proposed for detecting keystone taxa in microbial communities by examining each taxon's total influence on the rest of the community, without relying on pairwise interaction models or specific dynamics.^[^ ^44^ ^]^ Several phenotypes linked to microbiota composition have been shown to be heritable.^[^ ^45^, ^46^ ^]^ This suggests that host genetics may partially shape the microbiota, which in turn could influence the host's phenotype. Yang et al.^[^ ^26^ ^]^ investigated a quantitative trait locus involving a 2.3 kb deletion in *N*‐acetyl‐galactosaminyl‐transferase, altering *N*‐acetyl‐galactosamine levels and reducing *Erysipelotrichaceae* abundance. Furthermore, diet and environmental microbiota modulate the host genotype expression, especially during early developmental stages, when the immature gut microbiota is more susceptible to external influences.^[^ ^47^, ^48^, ^49^ ^]^ This emphasizes the need for effective characterization methods of maternal microbiota. Recently, LSTM has been widely used to analyzing complex microbial transmission dynamics. PhyLoSTM, combined convolutional neural networks and LSTM, was proposed to analyze longitudinal microbiome data, incorporating host environmental factors for disease prediction.^[^ ^50^ ^]^ Baranwal et al. developed and applied a LSTM framework to model the dynamics and functions of synthetic human gut microbiomes, outperforming traditional ecological models and enabling the design of microbiomes with tailored health‐relevant metabolite profiles and temporal behaviors.^[^ ^51^ ^]^

In this study, the use of the attention‐based LSTM model (MOMTM) allowed us to identify key maternal microbes involved in microbial transmission and their temporal influence on piglet gut microbiota. In comparison to traditional methods like FEAST and SourceTracker, which are commonly used to infer microbial source contributions, our deep learning model provides several distinct advantages. FEAST and SourceTracker algorithms primarily estimate the proportions of microbial sources using compositional data without accounting for temporal dynamics or host–microbe interactions explicitly. In our study, FEAST analysis indicated higher maternal microbial contributions in indigenous piglets on days 3 and 21 (Figure S1e, Supporting Information) but lacked the ability to capture the dynamic progression and detailed interactions of microbial taxa across developmental stages. By contrast, the deep‐learning‐based LSTM model employed in our study also predicted and confirmed the heightened maternal transmission in indigenous piglets specifically during days 3–7 and 14–21 of lactation (Figure 2d). This phenomenon may be attributed to maternal microbes rapidly occupying niches in the piglet gut immediately after birth. By day 14, the pioneering microbes have shaped the host gut environment, creating conditions that allow a broader range of maternal microbes to establish and exert their influence.^[^ ^52^ ^]^ By incorporating temporal sequencing and transmission centrality analyses, *Christensenellaceae R‐7* group as the primary driver of this transmission was identified at different developmental stages. This capability enables targeted interventions, such as GOS supplementation, to specifically enhance beneficial taxa like *Christensenellaceae* (Figure 3d).

The *Christensenellaceae R‐7* group exhibited high TC in both breeds, suggesting that it plays an essential role in maternal microbial transmission. The high heritability of *Christensenellaceae*, previously shown in the human gut microbiome^[^ ^53^ ^]^ and pig microbiome,^[^ ^26^ ^]^ further supports its role as a keystone taxon in maintaining microbial diversity and stability.

Since the 2011 work by Arumugam et al.,^[^ ^54^ ^]^ which characterized the human gut microbiome and introduced the concept of “enterotypes,” it has been established that human enterotypes are driven by the genera *Bacteroides*, *Prevotella*, and *Ruminococcus*, reflecting distinct microbial functions and metabolic pathways. Interestingly, pig samples also show a similar enterotype stratification, roughly into *Bacteroides*‐, *Prevotella*‐, and *Lactobacillus*‐dominated groups, with indigenous pigs being more likely to form a *Lactobacillus*‐dominated enterotype, highlighting a unique trend in their gut microbiome structure (Figure 1d and Figure S2a (Supporting Information)).

Microbiota‐targeted modulation has emerged as a promising approach in recent research, focusing on the manipulation of gut microbiota to improve health outcomes. Gordon and co‐workers investigated the use of a microbiota‐directed complementary food to improve growth in children with moderate acute malnutrition.^[^ ^55^ ^]^ The results provide insights into how modifying gut microbiota composition could be linked to physiological pathways related to growth and development. Maternal dietary interventions significantly impact offspring growth and microbiota development. In a study of 86 mother–neonate pairs, two maternal microbiota clusters emerged: *Prevotella*‐ and *Ruminococcus*‐dominated. Higher intake of fiber, omega‐3 fatty acids, and polyphenols was associated with the *Ruminococcus* cluster, affecting neonatal microbiota and body mass index (BMI) trajectories.^[^ ^56^ ^]^ Conversely, a low‐fiber maternal diet impaired early immune protection via the microbiome‐dependent Flt3L axis,^[^ ^57^ ^]^ while a high‐fiber diet alleviated behavioral disorders by modifying gut microbiota and neural development.^[^ ^58^ ^]^ Here, targeted maternal microbial intervention with GOS supplementation significantly influenced the composition of offspring enterotypes, promoting the formation of beneficial enterotypes dominated by *Lactobacillus* and *Christensenellaceae R‐7* groups. These enterotypes were associated with increased microbial diversity and TDF digestibility.

Goodrich et al.^[^ ^53^ ^]^ showed *Christensenellaceae* as the most heritable taxon in the human gut microbiome, influenced by host genetics and forming a co‐occurrence network with other heritable microbes, including methanogenic *Archaea*. Additionally, interacting taxa such as *NK4A214* and *UCG‐002* (*Oscillospiraceae*) also exhibited high TC during critical windows (Figure 3d and Figure S4k (Supporting Information)). Roswall et al.^[^ ^59^ ^]^ tracking the gut microbiota of 471 Swedish children from birth to age 5, also revealed that *Methanobrevibacter* and *Christensenellaceae* were late colonizers and did not reach adult levels by age 5, correlating with increased alpha diversity.

*Christensenellaceae*, particularly *C. minuta*, is associated with beneficial metabolic traits such as lower BMI, reduced serum lipids, and decreased visceral fat. *C. minuta* is often depleted in individuals with metabolic disorders including type 2 diabetes, obesity, and hypercholesterolemia.^[^ ^53^, ^60^ ^]^ The administration of *C. minuta* to pregnant mice led to increased *C. minuta* and elevated propionate concentrations in both dams and offspring. These findings align with previous studies that reported the ability of *C. minuta* to modulate host metabolism and alleviate metabolic disease.^[^ ^53^, ^61^ ^]^ However, metagenomic resequencing of low‐biomass weaned mouse feces lacks the depth and resolution to distinguish closely related *C. minuta* strains, while traditional isolation and strain‐specific polymerase chain reaction (PCR) approaches are hindered by low abundance and high genomic similarity. Furthermore, the absence of high‐quality reference genomes for mouse‐derived *C. minuta* strains precludes the design of species‐specific PCR primers capable of distinguishing between endogenous murine *C. minuta* proliferation versus colonization by the administered porcine‐derived strain. Consequently, it remains plausible that maternal *C. minuta* reshaped the dam's metabolite profile—such as bile acids or SCFAs—that were transferred via milk and, in turn, selectively enriched the offspring's endogenous *C. minuta* populations. Additionally, Although *C. minuta* DSM 22607 has been reported to produce high levels of acetate and moderate amounts of butyrate (an ≈5:1 ratio) without measurable propionate production,^[^ ^61^ ^]^ the *C. minuta* strain we isolated from NX pigs exhibited a robust propionate‐synthesis capability. Moreover, interactions between microbiota and mucins are essential for gut colonization and health.^[^ ^62^ ^]^ Notably, we observed significantly elevated propionate levels in offspring from *C. minuta*‐administered dams (Figure 5f). This finding carries particular weight as: i) our porcine‐derived strain possesses a complete propionate biosynthesis pathway (Figure 5g), and ii) this metabolic capability is absent in all previously characterized *C. minuta* strains (e.g., DSM 22607), which exclusively produce acetate. This functional evidence provides indirect support for direct maternal–offspring transmission. Future studies employing stable isotope tracing (¹^3^C‐labeled substrates), or engineered strain‐specific genetic markers will be required to conclusively discriminate between direct microbial transmission and metabolite‐mediated niche modulation mechanisms. Our data revealed that maternal supplementation with *C. minuta* significantly increased offspring colonic mucus production and secretion related gene expressions, supporting enhanced mucus secretion and gut barrier integrity (Figure 5i–k). This pattern resembles the mechanism by which *Akkermansia muciniphila* enhances host mucin secretion and barrier function through increased utilization of intestinal mucins.^[^ ^63^ ^]^ Wang et al.^[^ ^64^ ^]^ revealed that *Bacteroides thetaiotaomicron* produces propionate via the methylmalonyl‐CoA mutase pathway, which plays a crucial role in intestinal goblet cell differentiation and mucus barrier homeostasis. Ma et al.^[^ ^65^ ^]^ also explored how propionate stimulates *Muc2* mucin production in intestinal goblet cells by inducing hypoxia and selectively activating HIF‐2α. Mucin‐derived O‐glycans (particularly, GlcNAc) act as fermentable fibers in the gut, can promote SCF production and supporting mucosal immune homeostasis.^[^ ^66^ ^]^ Additionally, gut bacteria can utilize endogenous metabolites—particularly pyruvate and its potential precursors (cysteine and methionine)—to synthesize propionate through distinct metabolic routes.^[^ ^67^ ^]^ Upregulated metabolites were mainly involved bile acid biosynthesis and amino acid metabolism, whereas downregulated ones related to nucleotide, energy, and histamine metabolism. *C. minuta* influences host metabolism by producing novel 3‐O‐acyl‐cholic acids, secondary bile acids that inhibit the intestinal farnesoid X receptor.^[^ ^60^ ^]^ The increase in central carbon metabolism intermediates, such as malic acid in the tricarboxylic acid cycle, indicated altered energy metabolism demands.

In conclusion, our findings reinforce the concept that keystone species like *C. minuta* is integral to the establishment and maintenance of a healthy gut microbiome. By leveraging advanced machine learning models and targeted microbial interventions, this study lays the groundwork for novel strategies in microbiome management and therapeutic modulation, with potential applications extending to human health and disease prevention.

## 4 Experimental Section

### Animal Studies and Data Acquisition—Animal Trial 1

To comprehensively investigate maternal–offspring microbial transmission dynamics in pigs, extensive fecal sample collection from diverse pig breeds under controlled environmental conditions was conducted. A total of 892 fecal samples were collected from three pig lines including NX, Landrace × Yorkshire (LY), and Yorkshire (YKS). Specifically, 459 samples were obtained from 50 NX sow–piglet pairs at piglet days 3, 7, 21, 28, 90, and 150, as well as sow gestation day 112 and lactation days 3, 7, and 21. 310 samples were collected from 46 LY sow–piglet pairs at piglet days 3, 7, 14, 21, 28, 35, 90, and 150, and sow gestation day 112 and lactation days 7 and 21. 123 YKS samples were obtained at piglet days 3, 7, 14, 21 and sow lactation days 3 and 7. The study was conducted at the Yongxing Pig Farm of Hunan Chuweixiang Agriculture & Animal Husbandry Co., Ltd. (Changsha, China) and at Sifanghong farm (ZhangJiakou, China). In addition, 2018 sequencing samples were retrieved from public databases, including fecal samples of Mashen pigs (MS), JinFen (JF), Meishan pigs, and commercial pigs (Landrace × Large White (LLW), LY, Large White × Landrace (LWL)) involving both sows and piglets. In total, 2910 fecal microbiome datasets were compiled (sample distribution and proportions are illustrated in Figure 1a). All animal procedures were approved by the Institutional Animal Care and Use Committee of Hunan Agricultural University.

### Animal Studies and Data Acquisition—Animal Trial 2

To evaluate whether targeted maternal gut microbial interventions could alter offspring microbial functions, 36 multiparous NX sows (parity 3–5, with an average historical litter size of 9 piglets) were randomly assigned into three groups: a GOS supplementation group, an antibiotic treatment group, and a control group (Figure 3a). GOS supplementation was provided at 0.5% (70% purity) from gestation day 109 until weaning. The basal diet met the nutrient requirements outlined in NRC (2012) (see Table S2 in the Supporting Information). The antibiotic treatment group received chlortetracycline (100 ppm), ciprofloxacin (20 ppm), and vancomycin (10 ppm) in the same period. Cross‐fostering was carried out immediately postbirth, selecting piglets with an average birth weight of ≈950 g before colostrum feeding. Milk and vaginal introitus samples were collected from sows at parturition, while fecal samples were collected from sows at lactation days 7 and 14. Piglet fecal samples were collected on days 7, 14, and 21 of lactation. Following weaning and a 7 days adaptation period, piglets of five experimental groups were fed a high fiber diets formulated to meet NRC (2012) nutrient requirements (Table S3, Supporting Information).

Sows were transferred to individual farrowing pens one week before the expected delivery date. The farrowing rooms were maintained at a temperature of 20 °C, with a lighting schedule from 06:00 to 16:00. Sows were fed twice daily at 7:00 am and 3:00 pm following the farm's standard feeding protocol, with a diet formulated to meet the NRC 2012 nutrient requirements (Table S2, Supporting Information). Water was provided ad libitum.

### Animal Studies and Data Acquisition—Animal Trial 3

To investigate the functional changes of *Christensenellaceae* in piglets at different growth stages, a longitudinal study was conducted tracking 90 LY piglets from birth until 50 days of age. Fecal samples were collected at weekly intervals (total of seven sampling time points) and subjected to deep sequencing at 20G per sample.

All piglets were raised under uniform feeding and management conditions at Hunan Longhua Animal Husbandry Co., Ltd. They were provided ad libitum access to water throughout the experimental period. A standard commercial corn–soybean diet was used. Housing conditions, including temperature, humidity, and ventilation, were managed according to routine farm practices, and all other management procedures (e.g., vaccination, deworming) followed standard operating protocols.

### Animal Studies and Data Acquisition—Animal Trial 4

To validate whether *C. minuta* could influence offspring through maternal transmission, 18 4–5 weeks old female ICR mice were randomly assigned to two groups and housed under specific pathogen‐free conditions at the Hunan Agricultural University, Changsha, Hunan (Figure 5a). After one week of acclimation, mice were mated, and upon successful pairing, groups received oral gavage with 5 × 10⁸ colony‐forming units of *C. minuta* suspended in sterile phosphate‐buffered saline (PBS). Administration continued from mating through lactation until weaning. Fecal samples from dams were collected during late pregnancy and postweaning, while fecal samples from offspring were collected after weaning. *C. minuta* was cultured in peptone–yeast–glucose (PYG) medium at 37 °C prior to administration. All animal procedures were conducted in accordance with the Institutional Animal Care and Use Committee of Hunan Agricultural University (Changsha, Hunan, China).

### DNA Extraction and 16S rDNA Gene Sequencing

DNA was extracted using the TGuide S96 Magnetic Soil/Stool DNA Kit (Tiangen Biotech, Beijing) following the manufacturer's instructions. The V3–V4 region of the 16S rRNA gene was amplified using the universal primer pair 338F and 806R, with Illumina sample‐specific indices attached for sequencing. PCR amplicons were purified, quantified, and pooled for sequencing on an Illumina NovaSeq 6000 platform. Illumina sequencing and processing of sequencing data were performed by Beijing Biomaker Technology Co., Ltd. (China). Quality filtering, trimming, denoising, and amplicon sequence variant (ASV) construction were carried out using DADA2 (v1.16.0). Paired‐end reads were filtered (maxEE = 2, truncLen = 200/150 bp, truncQ = 2), denoised, merged (minOverlap = 12, maxMismatch = 0), and chimera sequences were removed.^[^ ^68^ ^]^ To ensure comparability across samples, rarefaction was performed to normalize sequencing depth to the minimum number of reads per sample using phyloseq R packages (v.1.46.0). Additionally, samples with low sequencing depth or potential contamination (e.g., outliers identified by extreme alpha diversity values or unusual taxonomic composition) were excluded from downstream analysis. Taxonomic classification of representative sequences was performed using Naïve Bayesian Classifier based on the Silva v138 reference database.

### Microbiome Data Preprocessing

Microbiome data were obtained from two primary sources: metadata and ASV tables. The metadata contained sample‐specific information, while the ASV data detailed microbial abundances across samples. To address the issue of zero counts in microbiome data, which could cause instability in the model due to data sparsity, the count zero multiplicative method was employed from the zCompositions (v.1.5.0‐4) R package, accessed through the rpy2 interface in Python (v.3.12.3), to appropriately replace zero values. The zCompositions package was employed for zero replacement using the central log‐ratio (CLR) transformation

(1) $$CLR\left(P\right)=\left(\log\frac{P_{1}}{g\left(P\right)},\log\frac{P_{2}}{g\left(P\right)},\text{…},\log\frac{P_{N}}{g\left(P\right)}\right)$$

where $g\left(P\right)={\left(\prod_{i=1}^{N}P_{i}\right)}^{1/N}$ is the geometric mean of the components of *P*. The dataset was divided into maternal (sow) and offspring (piglet) groups, further segmented into sucking and weaned phases based on developmental days.

The encoder processed the time series of maternal microbiota data to capture temporal dynamics and essential features influencing microbial transmission. Formally, for each sow, the encoder took as input the sequence ${\left\{P_{m}^{\left(t\right)}\right\}}_{t=1}^{T}$ and mapped it to a latent representation *H_m_*

(2) $$H_{m}=Encoder\left({\left\{P_{m}^{\left(t\right)}\right\}}_{t=1}^{T}\right)$$

The encoder was implemented using a LSTM network, which was well‐suited for modeling sequential data and capturing temporal dependencies.

The decoder predicted the piglet's microbiota compositions at time points, utilizing the latent representation *H_m_* and an attention mechanism to focus on the most relevant time points and microbial features

(3) $${\hat{P}}_{m}^{\left(t\right)}=Decoder\left(H_{m},t\right)$$

where ${\hat{P}}_{m}^{\left(t\right)}$ is the predicted piglet microbiota at time *t*.

The attention mechanism computed attention weights α_*t*_ that highlighted the contribution of microbial feature to the prediction

(4) $$\alpha_{t}=softmax\left(W_{a}\cdot H_{m}^{\left(t\right)}+b_{a}\right)$$

where *W_a_* and *b_a_* are learnable parameters, and $H_{m}^{\left(t\right)}$ is the hidden state at time *t*.

A composite loss function was designed that combined MSE, Euclidean loss, and L1regularization

(5) L=α×EuclideanLoss+β×MSE+λ×L1Regularization

where EuclideanLoss measures the root mean squared difference between predicted and actual piglet microbiota compositions, MSE is a sum of MSE at each time point *t*, L1Regularization encourages sparsity in the model parameters to prevent overfitting, *α*, *β*, and *λ* are the hyperparameters controlling the contributions of each component.

For each piglet, the trained model was used to predict the piglet's microbiota compositions ${\hat{P}}_{o}^{\left(t\right)}$ using the full maternal microbiota data. Each microbial taxon *i* was then systematically removed from the maternal microbiota data:

1. ${\hat{P}}_{m,i}^{\left(t\right)}=0$ was set for all *t*.
2. The maternal microbiota vectors $P_{m}^{\left(t\right)}$ were renormalized to ensure they remained in the probability simplex.
3. Using the modified maternal data, new predictions ${\hat{P}}_{m,i}^{\left(t,-i\right)}$ were generated.
4. The impact of removing taxon *i* was quantified by computing the TC between the original and modified predictions
(6) $$\Delta D_{i}=\frac{1}{T}\sum_{i=1}^{T}\left|{\hat{P}}_{o}^{\left(t\right)}-{\hat{P}}_{o}^{\left(t,-i\right)}\right|$$

### Tenfold Cross‑Validation

Model generalizability was evaluated using tenfold cross‐validation implemented with scikit‐learn (v1.2.2). Unique sow–piglet pair IDs were split into ten subsets via KFold(*n*_splits = 10, shuffle = True, random_state = 42) from sklearn.model_selection. In each fold, 90% of pairs formed the training set and 10% the validation set. For data batching, DataLoader from torch.utils.data (PyTorch v2.0.1) was used, ensuring shuffling at each epoch. After training the attention‐augmented LSTM encoder–decoder for 150 epochs with the Adam optimizer, fold‐level performance metrics—MSE, MAE, and *R* ^2^—was computed using mean_squared_error, mean_absolute_error, and r2**_**score from sklearn.metrics (v1.2.2). Finally, these scores were averaged across all tenfold to obtain the cross‐validation results.

### Metagenomics Sequencing for Gut Microbiota

A paired‐end library with an insertion size of 350 bp was constructed for 30 pig samples using the Illumina NovaSeq 6000 platform (Illumina, USA). High‐quality reads were obtained by filtering the raw sequencing data to remove adaptor sequences, low‐quality reads, and host genomic DNA contaminants. Specifically, the raw data were filtered using Trimmomatic (version v.0.33).^[^ ^69^ ^]^ Host contamination was removed by aligning the reads to the host reference genome using Bowtie2 (version v.2.2.4).^[^ ^70^ ^]^ The metagenome was assembled using MEGAHIT (version v.1.1.2)^[^ ^71^ ^]^ with default parameters, and contigs shorter than 500 bp were discarded. The quality of the assembled sequences was evaluated using QUAST (v.2.3)^[^ ^72^ ^]^ with default settings. Open reading frame prediction was performed using Prokka (version 1.11).^[^ ^73^ ^]^ To remove redundant sequences, CD‐HIT (version 4.8.1)^[^ ^74^ ^]^ was used for clustering with a sequence identity threshold of 90%.

Functional annotation of nonredundant protein sequences was performed using various databases and tools. HMMER (v.3.4) (http://hmmer.org/)^[^ ^75^ ^]^ and DIAMOND (v.2.0.15.153)^[^ ^76^ ^]^ (http://www.diamondsearch.org) were employed to identify carbohydrate‐active enzymes by comparing protein sequences against the CAZymes database (V.8).The protein sequences of the nonredundant genes were also annotated against the KEGG database using KofamScan (v.1.3.0). Based on the constructed gene profiles, Salmon was used to map the clean reads from each metagenome—retaining only those assigned to prokaryotes—against the nonredundant gene dataset. This approach yielded transcripts per million (TPM) values for each gene within every metagenome.^[^ ^77^ ^]^ These gene‐level TPMs were then aggregated by KEGG orthology (KO) pathways, deriving parts‐per‐million estimates (i.e., one KO‐pathway‐assigned sequence per million total sequences) for each metagenome.

### Genomic Binning, Genome Annotation, and Relative Abundance

Contigs were first processed with Fairy^[^ ^78^ ^]^ (v0.5.7) to rapidly estimate multisample coverage and then binned into MAGs using MetaBAT2^[^ ^79^ ^]^ (v2.12.1). Quality, gas chromatographic (GC) content, genome size, and taxonomy of the MAGs were assessed with CheckM^[^ ^80^ ^]^ (v1.0.12). MAGs were dereplicated using dRep^[^ ^81^ ^]^ (v3.2.0) with criteria of 95% ANI, a minimum completeness of 70%, and a maximum contamination of 10%. The MAG with the highest dRep score within each 95% ANI cluster (defined as a phylogroup) was selected as the representative for that group (Refseq: *C. hongkongensis* (GCA_004342745.1); *C. intestinibominis* (GCA_001678845.1); *C. massiliensis* (GCA_040409835.1); *C. minuta* (GCA_003628755.1); *C. tenuis* (GCA_014287795.1); *C. timonensis* (GCF_900087015.1)). Gene prediction was conducted with Prodigal (v2.6.3).

To identify function differences of maternal MAGs with the highest versus lowest prevalence in piglets, each sow bin was first matched to its corresponding piglet samples via the shared pair ID. For each bin, its prevalence at each stage was calculated as the fraction of piglet samples in which the bin's relative abundance was >0. These stage‐specific prevalences were then averaged to obtain an overall transmission prevalence for each bin. Bins were ranked by this mean prevalence, and the top 10% (highest‐prevalence) and bottom 10% (lowest‐prevalence) were selected for downstream analyses. Finally, functional annotation of nonredundant protein sequences from both bin subsets was performed following the same pipeline described in section “Metagenomics Sequencing for Gut Microbiota.”

### Construction of an Adhesin and QS Reference Database

An adhesin and QS reference database was constructed using protein sequences of adhesin‐related proteins and QS‐related proteins obtained from **N**CBI (https://www.ncbi.nlm.nih.gov/) and UniProt (https://www.uniprot.org/). These protein sequences, experimentally characterized in previous studies, were compiled and used as training sequences to establish the adhesin reference database.

Putative homologues of adhesin proteins in the metagenomes were identified using the DIAMOND and HMMER against the adhesin reference database. The following thresholds were applied during the search: sequence identity ≥ 30%, alignment coverage ≥ 50%, and *E*‐value ≤ 1e−5. All identified homologues were further analyzed using the Conserved Domain Database and the non‐redundant protein database from NCBI to confirm the presence of conserved domains. Sequences lacking conserved domains or annotations related to adhesin proteins were discarded.

### Isolation and Culture of *C. minuta* from Ningxiang Pigs

Fresh fecal samples were collected from an adult NX pig for the isolation of *C. minuta*. One gram of fecal sample was suspended in PBS and plated on PYG medium (Qingdao HopeBio, 37 °C for 2–7 days) to isolate *C. minuta*. Each single colony from each plate was purified on a new PYG plate, and bacterial colonies were sequenced using the primers F (5′‐ ACTCCTACGGGAGGCAGCA‐3′) and R (5′‐ GGACTACHVGGGTWTCTAAT‐3′). The 16S rRNA gene sequences were aligned to the NCBI nucleotide sequence database to confirm the identity of *C. minuta*. The isolated strain was stored at the Moon (Guangzhou) Biotec Co. Ltd. (MNA19939).

### Quantification of *C. minuta* by Quantitative Real‐Time PCR (qPCR)

The abundance of *C. minuta* was quantified by qPCR. DNA was extracted from mid‐colonic contents using a Stool DNA Isolation Kit according the manufacturer's instructions (SENO Biotech Co., Ltd., Zhangjiakou, China). DNA concentrations were measured fluorometrically using the Qubit dsDNA BR assay (Invitrogen) and standardized to 1 ng µL^−1^ for subsequent use as qPCR template.

Primers targeting the 16S rDNA gene of *C. minuta* (5′‐ AAGGAACGCAGGTGAGGTTT‐3′ and 5′‐ACGCTCATGAATCCCTTCCC‐3′) were employed for quantification. A standard curve was generated using nine concentrations ranging from 100 to 10⁸ gene copies per µL. Each qPCR reaction was performed in triplicate in a final volume of 10 µL, prepared with 500 nm primers and iQ SYBR green supermix (Bio‐Rad, USA), in 384‐well plates sealed with optical adhesive film. Amplification was conducted using a Bio‐Rad iCycler with the following conditions: initial denaturation at 95 °C for 10 min, followed by 40 cycles of 95 °C for 15 s, 60 °C for 20 s, and 72 °C for 30 s. A final denaturation step at 95 °C for 1 min was followed by a gradual increase in temperature from 60 to 95 °C (0.5 °C increments every 5 s) to generate a melt curve. Data analysis was conducted using Bio‐Rad CFX Manager 3.0 software. Copy number calculations were corrected based on the DNA concentration and the number of 16S rRNA gene copies present in the *C. minuta* genome.

### Whole‐Genome Analysis of *C. minuta*

PacBio sequencing data were processed using the SMRT Link tool to generate circular consensus sequencing (CCS) reads. Reads shorter than 2000 bp were filtered out to obtain high‐quality sequences for downstream analysis. The filtered CCS reads were assembled using Hifiasm software. The assembly was further corrected using Pilon software with Illumina data to improve accuracy. Assembled contigs were aligned against the NT database to determine the chromosome type. All processes were performed by biomarker Technologies (www.biomarker.com.cn) and BMKcloud (www.biocloud.net).

### SCFA Profiling Analysis

SCFAs in piglet cecal and colonic digesta, as well as ICR mouse colonic digesta, were analyzed using a GC system equipped with a flame ionization detector (Agilent 8890 series, Agilent Technologies, USA). Briefly, 1 g of each digesta sample was homogenized with 5 mL of deionized water, vortexed, and shaken on an oscillator for 30 min, followed by incubation at 4 °C overnight. After centrifugation at 10 000 × *g* for 10 min at 4 °C, the supernatant was collected, and the pellet was resuspended in 4 mL of deionized water and shaken for an additional 30 min. The total supernatant was subsequently centrifuged for 15 min at 12 000 × *g*.

The resulting supernatant was mixed with 25% metaphosphoric acid at a 9:1 volume ratio v/v and allowed to stand for 3–4 h at room temperature. If sediment appeared, the sample was centrifuged again at 12 000 × *g* for 15 min. The final supernatant was filtered through a 0.22 µm membrane filter and subjected to GC analysis. Chromatographic analysis was performed on a DB‐FFAP column (30 m × 250 µm × 0.25 µm; Agilent Technologies, USA) using high‐purity nitrogen as the carrier gas at a flow rate of 0.8 mL min^−1^. The injection volume was 1 µL, with a split ratio of 50:1, an injector temperature of 250 °C, and a detector temperature of 280 °C. The column temperature was initially held at 60 °C, then ramped to 220 °C at a rate of 20 °C min^−1^, and held for 1 min.

SCFAs, including acetic acid, propionic acid, butyric acid, isobutyric acid, isovaleric acid, and valeric acid, were identified based on retention times compared to reference standards. Standard curves were generated for quantification, and SCFA concentrations were calculated using Agilent OpenLab CDS 2.6 software to integrate peak areas. The concentrations of SCFAs were determined and normalized to the wet weight of the sample (mg/g).

### Metabolomics Analysis of Colonic Contents

A 50 µL aliquot of colonic content was homogenized with precooled methanol/water (4:1, v/v), vortexed for 5 min, and centrifuged at 12 000 × *g* at 4 °C for 10 min. The supernatant was dried using a vacuum centrifugal concentrator. The dried sample was reconstituted in acetonitrile/water (1:1) and centrifuged before analysis via ultrahigh liquid chromatography–mass spectrometry (UHPLC–MS; Q‐Exactive Plus, Thermo, USA). To ensure data quality, a pooled quality control (QC) sample was prepared by combining equal volumes of all experimental samples and analyzed alongside the study samples throughout the UHPLC–MS run sequence. The QC sample was injected every 10 samples to monitor instrument stability and reproducibility. Any samples showing significant deviation from the QC profile (e.g., >30% variation in peak intensity or retention time) were excluded from further analysis. To account for technical variability and batch effects, normalization was performed by scaling the intensity of each metabolite to the total ion current (TIC) of the corresponding sample. This TIC‐based normalization method adjusted for variations in sample preparation and instrument performance by dividing the peak area or intensity of each metabolite by the sum of all peak areas or intensities within the same sample. Orthogonal partial least squares discriminant analysis (OPLS‐DA) was performed using compound discoverer 3.3, with metabolite scaling via the Pareto method. Model validity was assessed by R2 and Q2, ensuring interpretability and predictability while minimizing overfitting. VIP scores were calculated to identify metabolites contributing most to group separation, with VIP >1 considered statistically significant. The VIP value for each metabolite was derived from the OPLS‐DA model using the formula

(7) $$VIP_{j}=\sqrt{p\frac{\sum_{a=1}^{A}\left(W_{ja}^{2}SS_{a}\right)}{\sum_{a=1}^{A}SS_{a}}}$$

where *p* represents the total number of metabolites, *A* denotes the number of principal components, and SS*_a_* corresponds to the sum of squares for the *a*‐th component. Metabolites with VIP scores exceeding 1.0 were considered to exhibit significant intergroup differences. For the identification of differentially abundant metabolites, the results were further integrated with FC values, applying a dual threshold of |log_2_FC| > 1 and VIP > 1 to ensure robust discrimination of biologically relevant metabolic alterations.

### Intestinal Tissue Section Preparation and Histomorphological Analysis

The fixed colonic tissues were sequentially dehydrated through an ethanol gradient (70%, 80%, and 90%, 30 min each), followed by two changes of 95% and absolute ethanol (20 min each). After complete dehydration, tissues were embedded in paraffin, sectioned, and dewaxed. The dewaxed sections were processed through xylene I and xylene II (10 min each), then rehydrated through a descending ethanol series (100%, 95%, 90%, 80%, and 70%, 5 min each) followed by a 1 min distilled water rinse.

For hematoxylin and eosin staining, sections were stained with hematoxylin for 5–10 min, rinsed under running water for 5 min, differentiated in 1% acid alcohol for several seconds, and blued in running water for 10–15 min. Counterstaining was performed with 0.5% eosin solution for 1–3 min. The stained sections were then rapidly dehydrated through an ascending ethanol series (70%, 80%, 90%, 95%, and 100%, 30 s to 1 min each), cleared in xylene (5 min × 2 changes), and mounted with neutral balsam. Histomorphological examination and quantitative analysis were performed using the CaseViewer whole‐slide imaging system (3DHISTECH, Hungary).

### Immunofluorescence Staining

Immunofluorescence double staining was performed to examine *Muc2* protein expression and nuclear localization in colonic tissues. Tissue section preparation followed the protocol described in Section “Intestinal Tissue Section Preparation and Histomorphological Analysis.” After washing with PBS, sections were blocked with 3% BSA for 30 min at room temperature, followed by incubation with primary rabbit anti‐*Muc2* antibody (1:200 dilution; Abcam, ab90007) at 4 °C overnight. The next day, after PBS washing, sections were incubated with CY3‐conjugated donkey anti‐rabbit IgG secondary antibody (1:500 dilution; Servicebio, GB21404) for 50 min at room temperature in the dark. Nuclei were counterstained with DAPI for 10 min before mounting with antifade mounting medium.

Fluorescent signals were visualized using a Nikon Eclipse C1 fluorescence microscope and captured with a Pannoramic MIDI scanner. *Muc2*‐positive signals appeared as red fluorescence (CY3: excitation 510–560 nm, emission 590 nm), while nuclei were visualized as blue fluorescence (DAPI: excitation 330–380 nm, emission 420 nm). Negative controls were included to ensure staining specificity, and all imaging parameters were kept constant throughout the experiment to maintain result comparability.

### Composition Analysis

Following dietary fiber intervention initiated at 28 days of age, fecal samples were collected from piglets for three consecutive days at both 35 and 45 days of age. Immediately after collection, each 100 g fecal sample was preserved with 10 mL of 10% dilute sulfuric acid for nitrogen fixation. The 3 days consecutive samples from each collection period were thoroughly homogenized and dried at 65 °C for 72 h. The dried samples were subsequently ground, sieved, and stored at −20 °C for compositional analysis. Feed and fecal ingredients were analyzed following the official methods of analysis of the AOAC International.^[^ ^82^ ^]^ Crude protein was measured using method 976.05, dry matter by method 930.15. TDF was determined with Auto Dietary Fiber Analyzer TDFi (Ankom Technology, Macedon, NY, USA).

### Strain‐Level of *C. minuta* Profiling by PanPhlAn

PanPhlAn,^[^ ^83^ ^]^ a strain‐level metagenomic profiling tool, was employed to identify the gene composition of *C. minuta* at different developmental stages. PanPhlAn used presence/absence patterns in the genomic content across members of the same species in complex metagenomic samples, allowing to characterize the dynamic gene profiles of *C. minuta* over time.

### Molecular Docking

The molecular structures of the ligands GalNAc‐Ser/Thr, GlcNAc‐Ser/Thr, Ser‐fucose, and pNP‐galactose were modeled using Discovery Studio (DS) 2019 (https://www.3ds.com/products/biovia/discovery‐studio) and subsequently optimized at the B3LYP/TZVP^[^ ^84^, ^85^ ^]^ theoretical level, incorporating Grimme's D3(BJ) empirical dispersion correction.^[^ ^86^, ^87^ ^]^ The geometric optimization convergence criteria were set as follows: i) the maximum and RMS forces on the nuclei were less than 0.00045 and 0.00030 Hartrees Bohr^−1^, respectively, and ii) the maximum and RMS nuclear displacements were less than 0.0018 and 0.0012 Å, respectively. These optimizations were performed using Gaussian16 (https://gaussian.com/gaussian16/).

Protein structures were modeled using AlphaFold 3.^[^ ^88^ ^]^ The highest‐ranking structure from the output was selected and protonated at pH 6.8^[^ ^89^ ^]^ to mimic the colonic pH environment of adult pigs via the H++ web server.^[^ ^90^ ^]^ All protein structures were further optimized in an explicit aqueous solution (OPC water model^[^ ^91^ ^]^) using the AMBER 19SB force field.^[^ ^92^ ^]^ The optimization protocol included 5000 steps of steepest descent followed by 5000 steps of conjugate gradient optimization, with a 100.0 kcal mol⁻¹ Å⁻^2^ restraint on the protein. Subsequently, the systems were gradually heated from 100 to 298 K in a 1 ns Langevin dynamics simulation under the canonical (*NVT*) ensemble, followed by an additional 1 ns relaxation in the isothermal–isobaric (*NPT*) ensemble using a Monte Carlo barostat^[^ ^93^ ^]^ for pressure control. The collision frequency was set to 1 ps⁻¹, and the time step was 1 fs. Simulations were carried out using AMBER22 (https://ambermd.org/). The last frame from each molecular dynamics simulation was collected for use in the docking studies.

The receptor–ligand Interaction module in DS 2019 was employed to identify potential ligand binding sites. Ligand–protein docking was performed using AutoDock Vina (https://vina.scripps.edu/), with the coordinates of the top‐ranked binding site serving as the center of the docking grid box. The grid box size was set to 30 × 30 × 30 Å, using the default grid spacing. The first predicted ligand–protein complex model was considered the near‐native conformation for analysis.

### TEM Preparation Method for *C. minuta*

TEM was used to visualize *C. minuta*. Bacterial cells were fixed with TEM fixative and postfixed in osmium tetroxide. After dehydration in an ethanol series, samples were embedded in 812 resin, polymerized, and sectioned into 60–80 nm ultrathin slices. Sections were stained with uranyl acetate and lead citrate, and imaged using a Hitachi HT7800 transmission electron microscope.

### Analysis of Gene Expression Levels

Total RNA was extracted from colonic tissues using RNAiso Plus reagent (Takara, Beijing). RNA concentration and purity were determined by a Nanodrop spectrophotometer (A260/A280 ratio), with all samples adjusted to a uniform concentration of 500 ng µL^−1^. First‐strand cDNA was synthesized from 1 µg of total RNA using the PrimeScript RT reagent kit (Takara) according to the manufacturer's protocol.

qPCR was performed on a LightCycler 480 II system (Roche, Germany) using SYBR Premix Ex Taq II (Takara), 10 µmol L^−1^ gene‐specific primers, and 50 ng cDNA template per reaction. The thermal cycling conditions consisted of an initial denaturation at 95 °C for 30 s, followed by 40 cycles of 95 °C for 5 s and 60 °C for 30 s. Melting curve analysis was subsequently performed to verify amplification specificity. Relative gene expression levels were calculated using the ΔCt method,^[^ ^94^ ^]^ with normalization to reference genes (β‐actin). All primer sequences are listed in Table S12 (Supporting Information), and each experiment included three technical replicates to ensure data reliability.

### Periodic Acid–Schiff (PAS) Staining

The distribution of glycogen and mucoproteins in colonic tissues was examined using PAS staining. Tissue section preparation followed the protocol described in Section “DNA Extraction and 16S rDNA Gene Sequencing.” Prior to staining, sections were dewaxed in eco‐friendly dewaxing solutions I and II (20 min each) and rehydrated through an ethanol gradient. The staining protocol consisted of: 1) oxidation with 0.5% periodic acid (PAS solution B) for 10–15 min, 2) Schiff reagent (PAS solution A) incubation for 25–30 min in the dark, 3) hematoxylin counterstaining for 30 s, followed by differentiation in acid alcohol and bluing in ammonia water. Sections were then dehydrated through an ascending ethanol series (70%, 80%, 95%, and 100%), cleared in xylene, and mounted with neutral balsam. Stained sections were examined under a Nikon Eclipse E100 light microscope, with digital images captured using a DS‐U3 imaging system for analysis. PAS‐positive substances (glycogen, mucoproteins, etc.) appeared magenta, while nuclei were stained blue.

### Statistical Analysis

All analyses were performed in R v4.3.3 within Rstudio v1.4.1717 or Python v3.8.13. All statistical tests were conducted using either parametric (one‐way analysis of variance with Tukey's post‐hoc test) or nonparametric (Kruskal–Wallis with Wilcoxon pairwise tests; ggpubr v0.6.0). Growth performance data were expressed as mean ± standard error of the mean (SEM). Pearson correlation coefficients (*r*) and their statistical significance (*p*‐values) were calculated using the scikit‐learn package in Python. For multivariate analysis, permutational multivariate analysis of variance (PERMANOVA) was conducted using the adonis2 function from the vegan package (v2.6‐4) in R, based on Euclidean distance matrices. The analysis generated pseudo‐F statistics with the corresponding *p*‐values to evaluate significant differences in microbial community composition.

The FEAST algorithm (v0.1.0) was used to estimate maternal microbial contributions, specifically assessing the influence of niches such as fecal, vaginal, and colostrum on piglet gut microbiota across developmental stages. Comparisons were made between related and randomized mother–piglet pairs, emphasizing the contribution from various maternal sources under different feeding conditions.

From the species composing the core microbiota with a detection threshold of 0.1% in feces and colon and 0.01% in mucosa in at least 50% of the samples, community profiles in these three sample types were estimated using DMM model fitting^[^ ^95^ ^]^ (*DirichletMultinomial* v.1.44.0 R package). Laplace approximation was used to evaluate DMM model fit, and the optimal number of components (clusters) was selected based on the lowest Laplace value. The main species driving differences between each community type were determined by selecting species with a cluster contribution values above the 80th percentile. The results obtained were visualized coloring samples in the ordination plots by cluster group and the differences on clusters were tested using a PERMANOVA analysis

Microbial diversity analyses were performed using R software (v4.3.3) with the vegan package (v2.6‐4). Alpha‐diversity was assessed using the Shannon diversity index and richness. Beta diversity was analyzed through PCA and NMDS based on Bray–Curtis dissimilarity matrices. The environmental fitting (envfit function) and PERMANOVA (adonis2 function) were employed to examine the relationships between microbial composition and experimental variables. All visualizations were generated using ggplot2 (v3.5.1).

Multiscale PHATE,^[^ ^96^ ^]^ an unsupervised dimensionality reduction technique, was employed to visualize the clustering and relationships between maternal and piglet microbiota. PHATE parameters included n_pca = 50, decay = 15, and knn = 20.

Microbiome enterotype transitions were visualized as Sankey diagrams in Python 3.9 using pandas (v1.5.3) for data manipulation and plotly (v5.9.0) for interactive plotting. First, per‐sample cluster assignments were imported with pd.read_csv() and filtered to the target cohort. Redundant observations at each pig_ID–stage combination were collapsed by taking the statistical mode, then pivoted to wide format so that each row corresponded to one pig and each column to one developmental stage. A predefined ordered list of stages was used to extract each pig's nonmissing stage sequence and generate source–target links labeled as “StageA_Cluster_*X*” → “StageB_Cluster_*Y*” with unit weight. Unique node labels were collected and indexed, and these indices fed into go. Sankey(node = dict(label = labels), link = dict(source = source_indices, target = target_indices, value = value)) to render the diagram.

Microbial co‐occurrence network analysis was performed using R software (v4.3.3) with the WGCNA (v1.72‐5) and igraph (v1.6.0) packages. The analysis began with preprocessing of ASV abundance data, retaining only features with mean relative abundance ≥0.5% across samples, followed by log10(*x* + 1) transformation to normalize distributions. Spearman rank correlations between ASVs were computed, with significant correlations (|*r*| ≥ 0.6, *p* < 0.001 after Benjamini–Hochberg correction) retained for network construction. The resulting adjacency matrix was imported into igraph for topological analysis, where four centrality measures were calculated for each node: degree centrality (normalized), betweenness centrality, closeness centrality, and eigenvector centrality. Network visualization employed the Fruchterman–Reingold force‐directed layout algorithm (9999 iterations), with node size scaled to degree centrality and edges colored by correlation direction (positive: gray; negative: red) and weighted by correlation strength. All analyses used set.seed(123) to ensure reproducibility, following established methodologies for microbial network analysis while incorporating robust statistical controls for multiple testing and data preprocessing.

Mantel analysis was performed using R software (v4.3.3) with the linkET (v1.0.3), vegan (v2.6‐4), and tidyverse (v1.3.1) packages. The analysis incorporated SCFA profiles from colonic contents and microbial abundance data, both processed to ensure sample alignment through row name matching. Mantel tests were conducted to assess correlations between individual SCFAs (acetate, propionate, isobutyrate, butyrate, isovalerate, and valerate) and microbial community composition, with results categorized by correlation strength (*r*: <0.2, 0.2–0.4, ≥0.4) and significance level (*p*: <0.01, 0.01–0.05, ≥0.05). Visualization was achieved through a lower triangular correlation heatmap using qcorrplot, incorporating Mantel test results as curved connectors between SCFAs and microbial taxa. The color gradient (RColorBrewer “RdBu” palette, 9 levels) represented Pearson correlation coefficients, while connector line properties (thickness and color) reflected Mantel *r* and *p* values, respectively.

To determine which oligosaccharide most effectively promotes the *Christensenellaceae R‐7* group, 16S rRNA gene sequencing data were retrieved from 216 oligosaccharide‐treated piglets (XOS, IMO, ASO, and GOS) deposited in NCBI. All raw reads were processed as described in Section “DNA Extraction and 16S rDNA Gene Sequencing” to generate an ASV abundance table. The randomForest package (v4.6‐14) in R was then used to fit a random forest regression model predicting the relative abundance of *Christensenellaceae R‐7* group from the four oligosaccharide treatments. The model was built with ntree = 1000 decision trees, and variable importance was assessed via the MeanDecreaseAccuracy (MDA) metric. Microbial features were systematically ranked according to their respective MDA values, where elevated MDA scores reflected: i) greater contribution to model predictive accuracy, and ii) stronger modulatory effects of the target oligosaccharide on the corresponding microbial taxa. The results indicated that GOS exerted the strongest regulatory effect on the *Christensenellaceae R‐7* group (Figure S4a and Table S1, Supporting Information).

## Conflict of Interest

The authors declare no conflict of interest.

## Author Contributions

H.S., X.M., and L.Z. contributed equally to this work. Y.Y., J.W., and B.T. designed the research. H.S., X.M., L.Z., H.L., and J.Z. conducted the experiments. H.S. and H.L. carried out bioinformatics and statistical analyses. K.Z. performed the molecular docking analyses. H.S. authored the paper. All authors reviewed and approved the final paper.

## Supporting information



Supporting Information

Supporting Information

## Data Availability

The data that support the findings of this study are available in the Supporting Information of this article. All 16S rRNA and metagenome sequencing data generated in this study have been deposited in the NCBI Sequence Read Archive (SRA) under accession numbers PRJNA1178342, PRJNA1210076 and PRJNA976087. Additionally, we utilized publicly available 16S rRNA and metagenome sequencing data from the SRA, including PRJEB53326, PRJNA682009, PRJNA733567, PRJNA721243, PRJNA718829, PRJNA913793, PRJNA302357, SRR24299145, PRJNA779404, PRJNA762151, SRP165134, PRJNA740238, PRJNA681971, PRJNA739889, PRJNA824854, PRJNA719480, PRJNA551340, and SRP049961, PRJNA373834, PRJNA779404, and PRJNA857725. Code used to process and analyse the data is available at https://github.com/shb289791170/MOMTM.
