# Supplementary material for: Targeted Intervention Strategies for Maternal–Offspring Transmission of Christensenellaceae in Pigs via a Deep Learning Model
Source: Adv Sci (Weinh). 2025 Jun 10;12(31):e03411. doi: 10.1002/advs.202503411 (PMC12376534; doi:10.1002/advs.202503411)
Supplement: Supplementary file 1 — Supporting Information [file ADVS-12-e03411-s002.docx]

# Targeted Intervention Strategies for Maternal–Offspring Transmission of Christensenellaceae in Pigs via a Deep Learning Model

Haibo Shen^a, b†^, Xiaokang Ma^a, b†^, Longlin Zhang^a, b†^, Hao Li^a, b^, Jichang Zheng^a, b^, Shengru Wu^c^, Ke Zuo^d^, Yulong Yin^b, e*^, Jing Wang^a, b*^, Bie Tan^a, b*^

^a^ *Key Laboratory of Hunan Province for the Products Quality Regulation of Livestock and Poultry, College of animal science and technology, Hunan Agricultural University, Changsha 410128, China.*

^b^ *Yuelushan Laboratory, Hunan, 410128, China.*

*^c^ College of Animal Science and Technology, Northwest A&F University, Yangling 712100, Shaanxi, China*

^d^ *National & Local Joint Engineering Research Center of Targeted and Innovative Therapeutics, Chongqing Key Laboratory of Kinase Modulators as Innovative Medicine, College of Pharmacy (International Academy of Targeted Therapeutics and Innovation), Chongqing University of Arts and Sciences, Chongqing, 402160, China.*

^e^ *Laboratory of Animal Nutritional Physiology and Metabolic Process, Key Laboratory of Agro-ecological Processes in Subtropical Region, National Engineering Laboratory for Pollution Control and Waste Utilization in Livestock and Poultry Production,* *Institute of Subtropical Agriculture, Chinese Academy of Sciences, Changsha 410125, China.*

† Contributed equally to this work

* Correspondence authors (Yulong Yin, yinyulong@isa.ac.cn; Jing Wang, jingwang023@hunau.edu.cn; Bie Tan, bietan@hunau.edu.cn)





**Fig. S1** (a) Phylum-level comparison of gut microbiota between Chinese indigenous (left panel) and commercial pig (right panel) from birth to day 150. Scatter plots depicting the Shannon diversity index (b) and Richness (c) (alpha diversity measures) ( Commercial: day1 n=72, day3 n=56, day7 n=226, day14 n=269, day21 n=221, day28 n=100, day35 n=181, day90 n=43, day150 n=16; Chinese indigenous: day1 n=6, day3 n=58, day7 n=64, day14 n=20, day21 n=57, day28 n=44, day35 n=12, day90 n=49, day150 n=39), Prevalance of maternal microbiota in piglet (d) (Commercial: day1 n=101, day3 n=62, day7 n=253, day14 n=322, day21 n=248, day28 n=118, day35 n=243, day90 n=44, day150 n=79; Chinese indigenous: day1 n=23, day3 n=65, day7 n=72, day14 n=39, day21 n=48, day28 n=50, day35 n=27, day90 n=40, day150 n=29) and Bray–Curtis dissimilarity (beta diversity measure) (f) of piglet fecal microbiota over time for both Chinese indigenous and commercial pigs (Commercial: day1 n=287, day3 n=197, day7 n=661, day14 n=593, day21 n=471, day28 n=317, day35 n=345, day90 n=256, day150 n=424; Chinese indigenous: day1 n=29, day3 n=134, day7 n=147, day14 n=39, day21 n=143, day28 n=130, day35 n=58, day90 n=156, day150 n=106). (e)FEAST (Fast Expectation-maximization for microbial Source Tracking) analysis estimates the contribution of maternal microbiota to piglet gut microbiota at different time points (Commercial: day1 n=101, day3 n=62, day7 n=253, day14 n=322, day21 n=248, day28 n=118, day35 n=243, day90 n=44, day150 n=79; Chinese indigenous: day1 n=23, day3 n=65, day7 n=72, day14 n=39, day21 n=48, day28 n=50, day35 n=27, day90 n=40, day150 n=29).. The Shannon index (g) and richness (h) of different enterotypes were compared between Chinese indigenous (yellow) and commercial (green) pig (Commercial: Enterotype1 n=234, Enterotype2 n=177, Enterotype3 n=103, Enterotype4 n=120, Enterotype5 n=134, Enterotype6 n=162, Enterotype7 n=177, Enterotype8 n=136, Enterotype9 n=154, Enterotype10 n=118, Enterotype11 n=92, Enterotype12 n=114, Enterotype13 n=81, Enterotype14 n=93, Enterotype15 n=19. Chinese indigenous: Enterotype1 n=32, Enterotype2 n=64, Enterotype3 n=98, Enterotype4 n=76, Enterotype5 n=51, Enterotype6 n=34, Enterotype7 n=16, Enterotype8 n=20, Enterotype9 n=7, Enterotype10 n=38, Enterotype11 n=45, Enterotype12 n=3, Enterotype13 n=27, Enterotype14 n=34.). Statistical comparisons were performed using Wilcox tests.


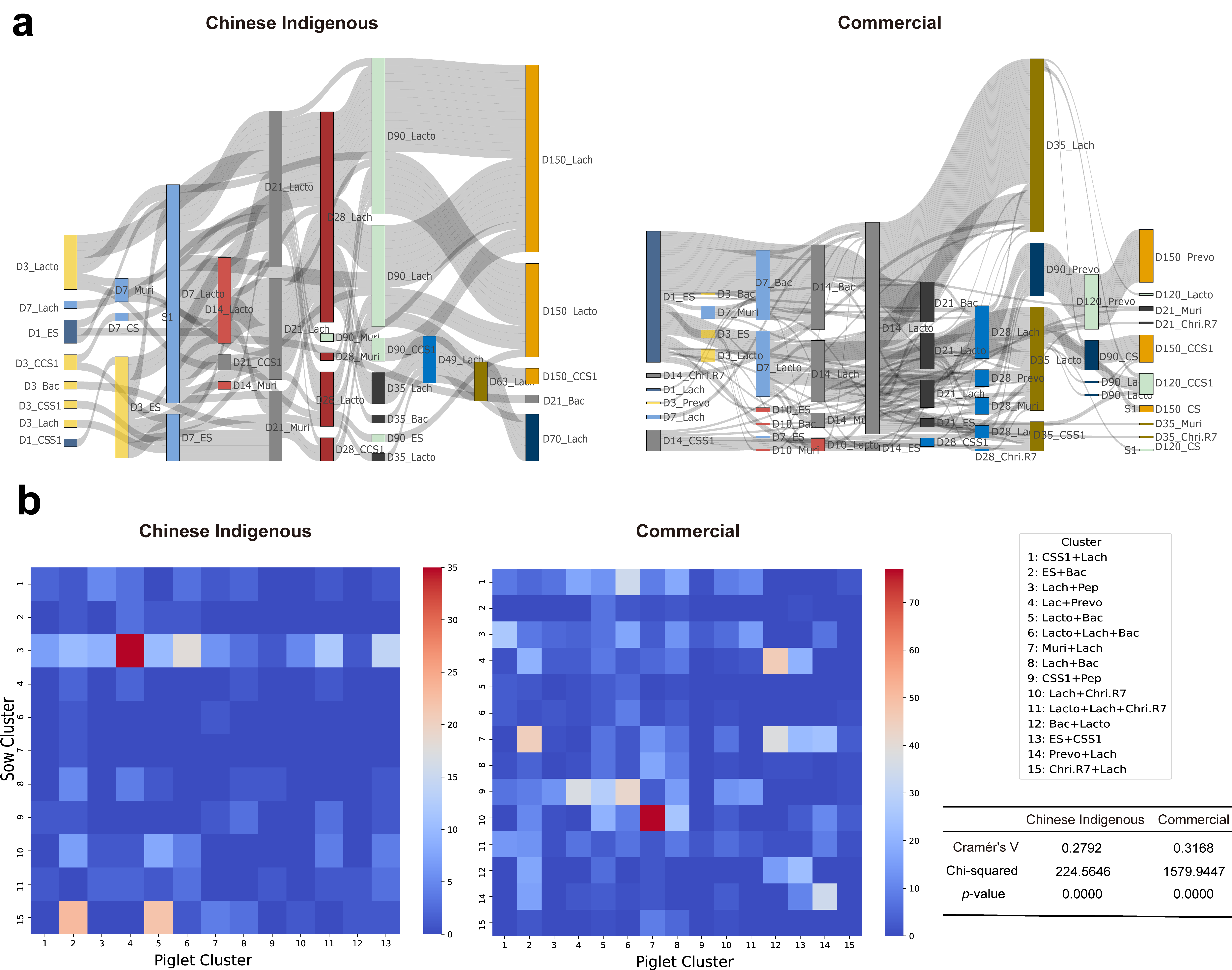


**Fig. S2** **Enterotype Dynamics and Maternal-Offspring Correlations in Chinese Indigenous and Commercial Pigs.** (a) Sankey diagrams illustrating enterotype transitions in Chinese indigenous (left panel) and commercial pigs (right panel) across various stages of lactation and growth. The thickness of the flows indicates the proportion of individuals transitioning between different enterotypes over time. (b) Heatmaps showing contingency analysis between sow clusters and piglet clusters for Chinese indigenous (left) and commercial pigs (right). The intensity of color represents the frequency of specific sow-piglet cluster associations. *Chri.R7* denotes the *Christensenellaceae_R-7* *group*; *CSS1* refers to *Clostridium sensu stricto 1*; *Lach* stands for *Lachnospiraceae*; *ES* represents *Escherichia-Shigella*; *Bac* indicates *Bacteroides*; *Prevo* signifies *Prevotella*; and *Lacto* corresponds to *Lactobacillus*.


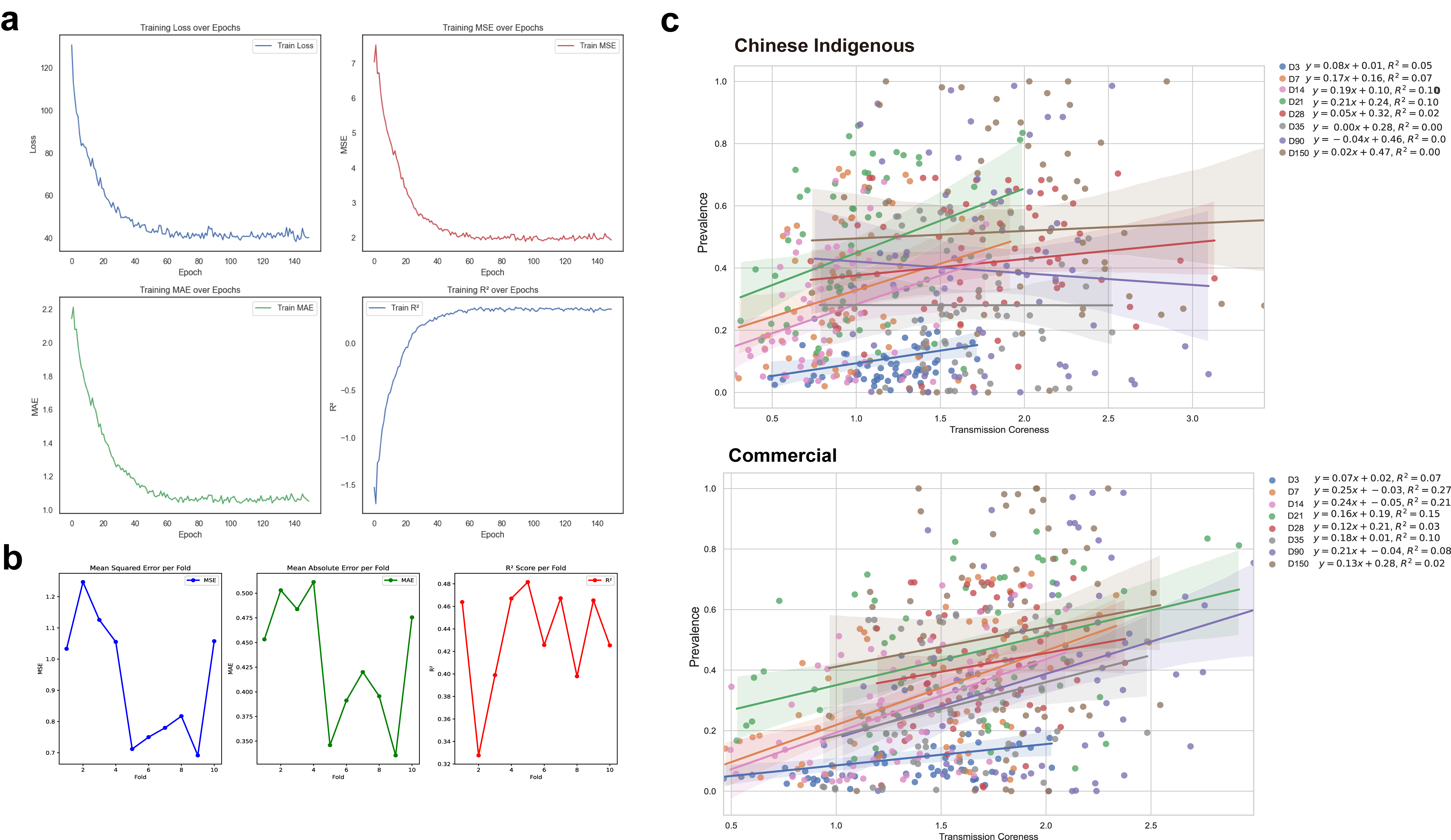


**Fig. S3** **Training Performance, Cross-Validation, and Transmission Correlation Analysis of the Maternal-Offspring Microbial Transmission Model (MOMTM).** (a)Model training performance of the Maternal-Offspring Microbial Transmission Model (MOMTM). Metrics including Mean Squared Error (MSE), Mean Absolute Error (MAE), and coefficient of determination (𝑅2) across 150 epochs of training are displayed. (b) Ten-fold cross-validation results (c) Correlation between transmission centrality and maternal positive bacteria prevalence in piglets.





**Fig. S4** **Impact of Oligosaccharides and Antibiotic Interventions on Microbial Composition and Enterotype Clustering.** (a) Random forest analysis of oligosaccharide impact. Mean Decrease Accuracy scores from random forest analysis assessing the influence of four oligosaccharides—xylo-oligosaccharides (XOS), arabinose-based oligosaccharides (ASO), isomalto-oligosaccharides (IMO), and galacto-oligosaccharides (GOS)—on the abundance of various bacterial genera. (b) DMM enterotype clustering of maternal colostrum and vaginal swabs of sows after GOS and antibiotic (AB) interventions. (c) Stacked bar plots representing the relative abundance (%) of bacterial phyla in colostrum (left) and vaginal introitus (right).(d) Microbial community dissimilarity between piglet gut microbiota from non-kin and kin sows during lactation at days 7, 14, and 21(Sow colostrum vs. day 7 piglet feces; Sow vagina vs. day 7 piglet feces; Sow feces (day 7) vs. piglet feces (day 7); Sow feces (day 14) vs. piglet feces (days 14 and 21)). (e) Temporal dynamics of Bray-Curtis dissimilarity between maternal and offspring microbiota across treatment groups. Box plots showing Shannon diversity index and microbial richness (f) in piglet fecal samples at days 7, 14, and 21 of lactation. Sample sizes were as follows: Control (n = 12, 13, 14), GOS (n = 10, 12, 14), AB (n = 12, 12, 12), AB-GOS (n = 11, 10, 11), GOS-AB (n = 18, 18, 15) at days 7, 14, and 21, respectively. Short-chain fatty acid (SCFA) including acetate, propionate, isobutyrate, butyrate, isovalerate, and valerate concentrations in colon (g) and cecal (h) chyme of piglets at day 21 (n = 7 per group). (i) Mantel correlation analysis was used to identify significant associations between SCFA concentrations and relative abundances of microbial composition in piglets. The heatmap displays the pairwise Pearson correlation coefficients between different bacterial genera. Positive correlations are indicated in blue, while negative correlations are shown in red, with the intensity of the color reflecting the strength of the correlation. Network edges represent significant correlations (adjusted P ≤ 0.05, |r| ≥ 0.2). (j) Network analysis depicting interactions among microbial taxa in the gut microbiota. Nodes represent taxa, and edges represent significant co-occurrence relationships. Heatmap displaying the differential abundance of (k)carbohydrate-active enzymes (CAZymes) and (l)KEGG pathway in piglet microbiota across treatment groups. (m) Histological examination of crypt depths among different treatment groups at the end of lactation. Scale bar: 200 μm. Error bars represent mean ± SEM. Multiple group comparisons were conducted using the Kruskal-Wallis test, followed by Dunn's post-hoc test with Benjamini-Hochberg correction for multiple comparisons.





**Fig. S5** **Functional Characterization of High- and Low-Prevalence Bins and *Christensenella* Species Across Developmental Stages.** (a) Phylogenetic tree of MAGs. A maximum-likelihood phylogenetic tree constructed from 2,202 medium-quality metagenome-assembled genomes (MAGs) (completeness > 70%, contamination < 10%). Circular charts representing KEGG pathway functional enrichment(b), carbohydrate-active enzymes (CAZymes) (c), adhesion-related genes (d), and quorum sensing genes (e) for the top 10% (yellow) and bottom 10% (blue) most prevalent bins during lactation and weaning. The size of the circle represents the relative abundance of a specific functional category. (f) Scatter plot comparing the abundance of specific EC enzyme classes between the top 10% (yellow) and bottom 10% (blue) most prevalent bins across different lactation and weaning stages. Each point represents the abundance value for a particular sample at the indicated stage, with point size reflecting the sampling stage. (g)Relative Abundance and Prevalence Profiles of *Christensenella* Species. (h)Mucin-Degrading Enzyme Profiles of *Christensenella* Species. *p* values < 0.05 were considered statistically significant (* *p* < 0.05, ** *p* < 0.01, *** *p* < 0.001).

**

**

**Fig. S6** **Phylogenetic, Structural, and Functional Characterization of *C. minuta* Isolated from NX Pigs.** (a)Phylogenetic Tree of *Christensenella* Strains. Circular phylogenetic tree displaying the relationships among various strains of the Christensenellaceae family, including *C. hongkongensis*, *C. intestinihominis*, *C. massiliensis*, *C. minuta*, *C. timonensis*, and *C. tenuis*, among others. Taxa from different sources are highlighted with specific markers: metagenome-assembled genomes (MAG) from pigs (star), *C. minuta* isolated from NX pigs (star), and sequences from the NCBI database (blue). (b) Transmission electron microscopy (TEM) image showing the cellular morphology of *C. minuta*. The scale bar represents 500 μm. (c)Whole-genome sequencing and functional annotation of *C. minuta* isolated from NX pig feces. (d) KEGG pathway enrichment analysis of *C. minuta*. The pathways are grouped into categories such as Cellular Processes, Metabolism, Environmental Information Processing, and Genetic Information Processing. (e) Phylogenetic Analysis of GH109 Enzymes (red) in *C. minuta* and other GH109 enzymes from NCBI. (f) KEGG pathway of propionate biosynthesis genes in *C. minuta* (Blue boxes indicate detected genes in the propionate synthesis pathway(see Table S3 for gene annotations)). (g) Non-metric multidimensional scaling (NMDS) plot showing differences in microbial communities between mothers and offspring. Samples are colored by treatment group: *C. minuta* (Cm, orange) and Control (Con, blue). Symbols differentiate mothers (circles) and offspring (crosses). The dotted lines connect paired mother-offspring samples, illustrating their relationship in community composition. (h) Shannon diversity and richness indices in fecal microbiota of dams and offspring in the control and *C. minuta* groups. (i) Relative abundance of the *Christensenellaceae R-7 group*, *NK4A214 group*, and *UCG-005_Oscillospiraceae* in dams and offspring. Multiple comparisons were performed using Kruskal-Wallis with dunn tests followed by Benjamini-Hochberg correction.
